# π‐Extended Polyaromatic Hydrocarbons by Sustainable Alkyne Annulations through Double C−H/N−H Activation

**DOI:** 10.1002/chem.201905023

**Published:** 2019-12-09

**Authors:** Elżbieta Gońka, Long Yang, Ralf Steinbock, Fabio Pesciaioli, Rositha Kuniyil, Lutz Ackermann

**Affiliations:** ^1^ Institut für Organische und Biomolekulare Chemie Georg-August-Universität Göttingen Tammannstraße 2 37077 Göttingen Germany

**Keywords:** annulation, C−H activation, diketopyrrolopyrroles, polyaromatic hydrocarbons, ruthenium

## Abstract

The widespread applications of substituted diketopyrrolopyrroles (DPPs) call for the development of efficient methods for their modular assembly. Herein, we present a π‐expansion strategy for polyaromatic hydrocarbons (PAHs) by C−H activation in a sustainable fashion. Thus, twofold C−H/N‐H activations were accomplished by versatile ruthenium(II)carboxylate catalysis, providing step‐economical access to diversely decorated fluorogenic DPPs that was merged with late‐stage palladium‐catalyzed C−H arylation on the thus‐assembled DPP motif.

## Graphical abstract

**PAHs**: Double C−H/N−H activation by ruthenium(II) catalysis enabled the assembly of π‐expanded diketopyrrolopyrroles with unique spectroscopic features (see scheme).

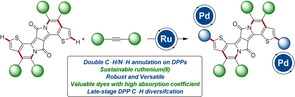

Since their first synthesis,^1^ diketopyrrolopyrroles (DPPs) have attracted great attention from researchers from various research arenas, including optoelectronic material sciences^2^ and bioimaging.^3^ These inconspicuous small organic DPP molecules exhibit versatile properties ranging from low solubility, chemical resistance, outstanding stabilities and distinct colors, which render them excellent pigments.^4^ *N*‐Alkylation leads to DPPs with improved solubilities, the properties of which can be fine‐tuned by the incorporation of different aromatic motifs.^3^ Due to their strong electron‐withdrawing ability and optical properties, DPPs are widely used as small molecules and conjugated semiconducting polymers in organic solar cells (OSCs),2a–2e, 2g, 5 organic field‐effect transistors (OFET),2f, 6 organic photovoltaic cells (OPVs),^7^ as well as fluorescent probes,^3^, ^8^ photocatalysts,^9^ photosensitizers^10^ or photothermal therapy agents,^11^ promising annihilator molecules,^12^ self‐assembled dyes,^13^ and bioconjugated hybrids.^14^

DPP derivatives with a highly decorated periphery as well as DPP‐based polymers are usually obtained through conventional condensation reactions4b, 15 or metal‐catalyzed cross‐coupling reactions with prefunctionalized substrates.4a, 8b, 16 In comparison to the elegant efforts devoted to the modification of the DPP periphery by direct functionalization or direct arylation polymerization (DArP),^17^ their de novo assembly leading to π‐extended structures continuous to be underdeveloped, with notable recent progress by Zumbusch^18^ and Würthner.^13^, ^19^ Another possibility is represented by the introduction of an ethylene bridge between the nitrogen and the aromatic moiety, which leads to significantly π‐expanded compounds.^20^ These DPP derivatives possess sharp absorption and emission peaks, with very high molar absorption coefficients and unique fluorescence quantum yields,20b featuring after further modification a two‐photon absorption cross‐section.20a Very recently, π‐extended DPP moieties were incorporated into alternating donor–acceptor copolymers resulting in a variety of low band gap copolymers.^21^ Despite of these indisputable advances, thus far, these DPPs were obtained by classical N‐alkylation with bromoacetaldehyde diethyl acetal, along with electrophilic aromatic substitution. Notably, this approach is hence limited to the introduction of ethylene junctions with only one substituent.

During the past decade, C−H activation has been identified as a transformative platform for various small molecule derivatizations through alkyne annulations.^22^ In sharp contrast, DPP assembly and diversification by C−H functionalization or DArP have thus far largely proven elusive.^17^ Within our program on sustainable C−H activation,^23^, ^24^ we hence devised a versatile strategy for the modular assembly of DPPs in a single step by twofold C−H/N−H activation (Figure 1). Salient features of our findings include: 1) innovative ruthenium‐catalyzed double C−H/N−H annulation of DPPs, 2) a π‐expansion‐strategy for PAHs, 3) access to valuable dyes with high absorption coefficients, and 4) transformative late‐stage diversification by C−H arylation on the DPP motif.

**Figure 1:**
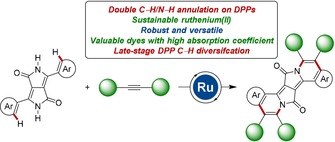
Double C−H/N−H activation for modular assembly of diketopyrrolopyrole PAHs.

We initiated our studies by probing various reaction conditions for the envisioned double C−H/N−H activation of diketopyrrolopyrrole **1 a** with alkyne **2 a** (Table 1, for detailed information, see Tables S1 and S2 in the Supporting Information).^25^ Thus, moisture‐ and air‐stable ruthenium(II) complexes emerged as the catalysts of choice, with most effective catalysis accomplished by carboxylate assistance (Table 1, entries 1–4).^26^ Given the rather poor solubility of substrate **1 a** 1, 4b and the aromatic character of PAH **3 aa**, we next explored various solvents (entries 4–9), with *o*‐xylene being superior. Control experiments confirmed the essential role of carboxylate‐assisted ruthenium(II) catalysis (entries 10–11). The particularly challenging character of the twofold C−H/N−H activation was reflected by palladium and iridium catalysts failing short in delivering the desired product **3 aa** (entries 13 and 14). Likewise {Cp*Rh^III^}‐catalysis was significantly less effective than the cost‐effective ruthenium(II) manifold (entries 15 and 16).

**Table 1:** Optimization of double C−H/N−H activation on DPP **1 a**.^[a]^

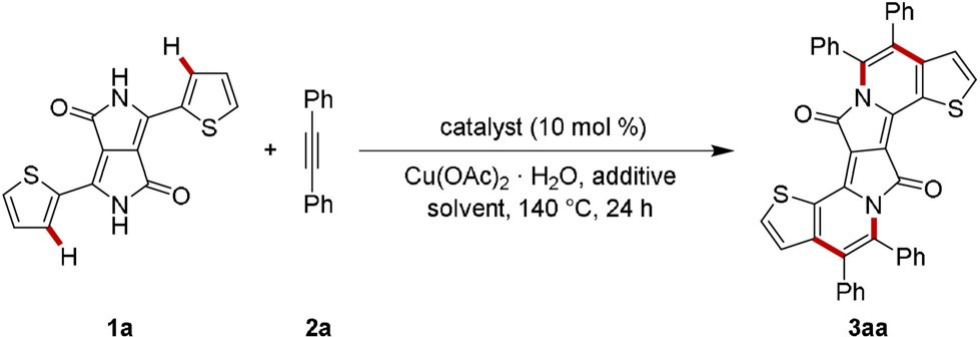				
Entry	Catalyst	Additive	Solvent	Yield [%]^[b]^
1	[RuCl_2_(*p‐*cymene)]_2_	–	*o‐*xylene	35
2	[RuCl_2_(*p‐*cymene)]_2_	KOAc	*o‐*xylene	80
**3^[c,d]^**	**[RuCl_2_(*p‐*cymene)]_2_**	**KOAc**	***o‐*** **xylene**	**92**
**4^[d]^**	**[RuCl_2_(*p‐*cymene)]_2_**	**KOAc**	***o‐*** **xylene**	**84**
5	[RuCl_2_(*p‐*cymene)]_2_	KOAc	DCE	70
6	[RuCl_2_(*p‐*cymene)]_2_	KOAc	PhMe	56
7	[RuCl_2_(*p‐*cymene)]_2_	KOAc	*t*AmOH	57
8	[RuCl_2_(*p‐*cymene)]_2_	KOAc	DMF	–
9	[RuCl_2_(*p‐*cymene)]_2_	KOAc	GVL	–
10	–	KOAc	*o‐*xylene	–
11^[e]^	[RuCl_2_(*p‐*cymene)]_2_	–	*o‐*xylene	–
12^[e]^	[RuCl_2_(*p‐*cymene)]_2_	KOAc	*o‐*xylene	–
13^[f]^	Pd(OAc)_2_	KOAc	*o‐*xylene	–
14	[Cp*IrCl_2_]_2_	KOAc	*o‐*xylene	–
15	[RhCp*Cl_2_]_2_	KOAc	*o‐*xylene	76
16^[c,d]^	[RhCp*Cl_2_]_2_	KOAc	*o‐*xylene	20

[a] Reaction conditions: **1 a** (0.25 mmol), **2 a** (1.00 mmol), catalyst (10 mol %), Cu(OAc)_2_ **⋅**H_2_O (0.5 mmol), additive (0.25 mmol), solvent (0.2 m), 140 °C, 24 h. [b] Isolated yields. [c] 16 h. [d] 100 °C. [e] CuBr_2_ as an oxidant. [f] 20 mol %.

With the optimized catalyst in hand, we tested its versatility in the double C−H/N−H activation of DPP **1 a** with a variety of aryl‐substituted alkynes **2** (Scheme 1). Thereby, π‐extended PAHs were accessed from electron‐rich as well as electron‐deficient alkynes in an efficient manner, including sensitive tetra‐bromo DPP **3 ag** and thiophene‐rich **3 ah**, which should prove instrumental for further modifications and applications of the DPPs **3**.

**Scheme 1:**
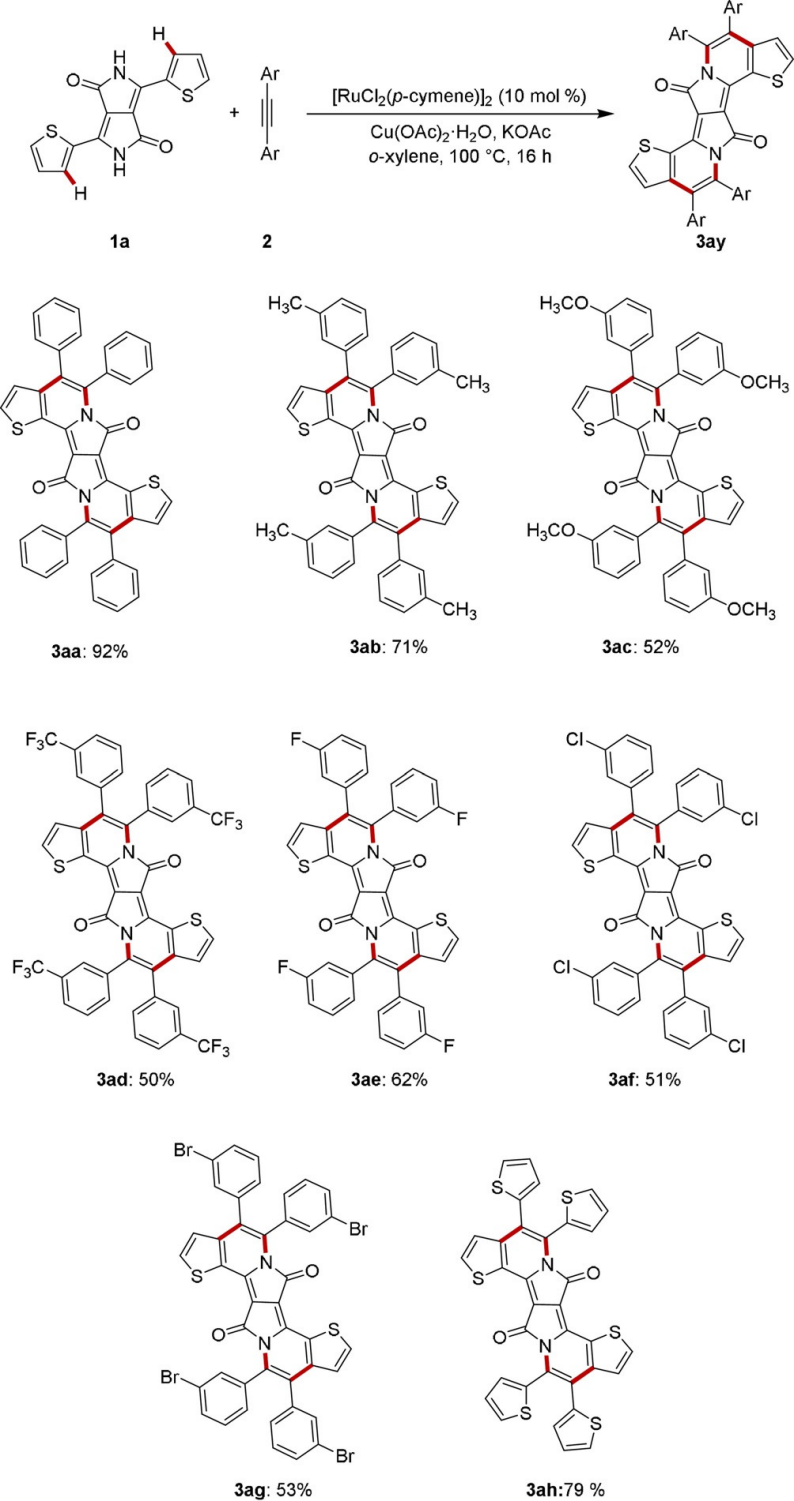
Ruthenium(II)‐catalyzed double C−H/N−H activation with aryl alkynes **2**.

The double ruthenium‐catalyzed DPP C−H/N−H activation was not restricted to unsubstituted, parent DPP **1 a** (Scheme 2). Indeed, the reaction also proceeded efficiently with alkyl‐substituted derivative **1 b** as well as the aryl‐modified‐DPP **1 c**, with the isolated yield of annulation product **3 ca** being caused by the extremely low solubility of substrate **1 c**.

**Scheme 2:**
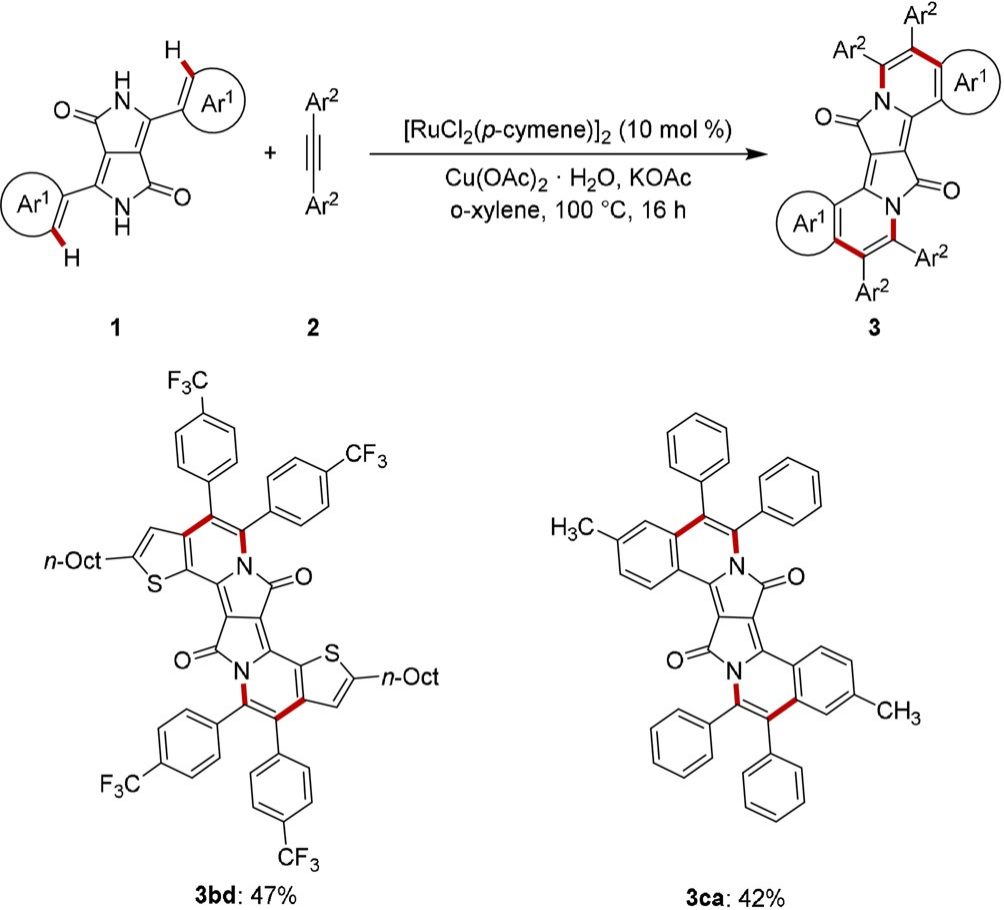
Double ruthenium‐catalyzed C−H/N−H activations of DPPs **1**.

Next, we evaluated alkyl‐substituted alkynes **4** in the twofold ruthenium‐catalyzed alkyne annulation by DPP **1 a** (Scheme 3). Hence, the desired π‐extended PAHs **5 aa** and **5 ab** were obtained by efficient oxidative C−H/N−H activations.

**Scheme 3:**
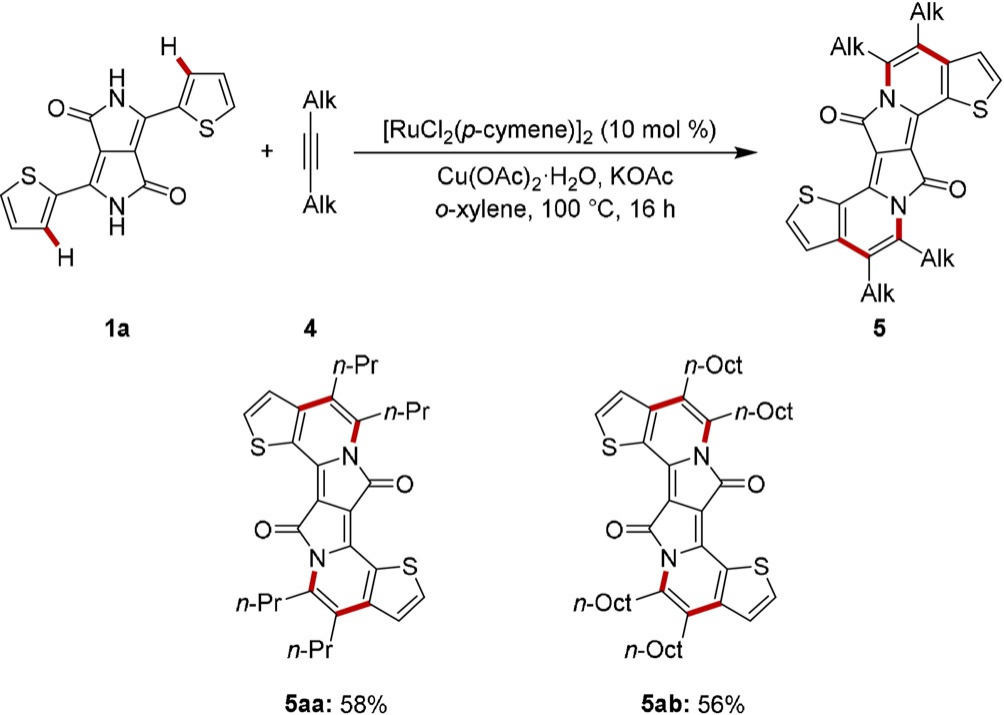
Twofold annulations of alkyl‐alkynes **4** by DPP **1 a**.

The connectivity of the annulated product **3 aa** was unambiguously established by X‐ray crystal diffraction analysis (Figure 2). The dihedral angle between the DPP unit and its adjacent thiophene unit was found with 6.4°, clearly showing the importance of the molecular tether to induce planarity, as compared with the unbridged compound.6c Further, the individual molecules are arranged in a lamellar packing motif,^27^ which appears to be stabilized by dispersive non‐covalent C−H⋅⋅⋅π interaction between the C−H bonds of the arene motif and the thiophene unit (2.82 Å).

**Figure 2:**
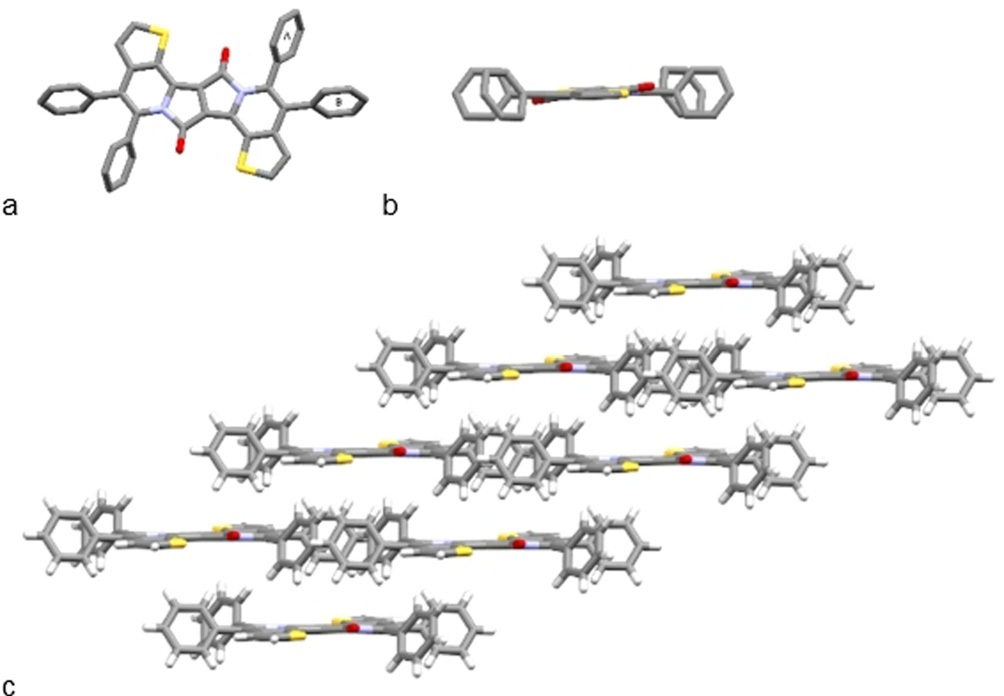
X‐ray structure of DPP **3 aa**. a) Molecular structure. b) Side view on the molecular structure, highlighting the planarity of the DPP core. c) Lamellar packing motif. Hydrogen atoms are partially omitted for clarity.

The optical properties of the thus‐obtained novel DPP PAHs **3**, **5** and **7** were thereafter studied by detailed UV/Vis absorption and fluorescence spectroscopy (Table 2). The unprecedented DPPs exhibited very intense absorption in the UV and visible region, with absorption maxima between maxima between 600–680 nm OR maxima between 600–640 nm for the annulation products **3** and **5**, which results in an intense blue to purple color. The absorption maximum in all synthesized derivatives is bathochromically shifted in comparison with the previously synthesized, unsubstituted compounds,20b whereas both Stoke shift and absorption coefficient are comparable. Interestingly, the highest absorption coefficients were obtained for the *p*‐tolyl‐DPP derivative **3 ca**, while the largest Stokes shift was observed for thiophene‐DPP derivative **3 aa** and **7**.

**Table 2:** Spectroscopic data of DPPs **3**, **5**, and **7**.

Compd.	*λ*_abs.max_	*λ*_em.max_	Stokes shift [cm^−1^]	*ϵ*_max_ [m ^−1^ cm^−1^]
**3 aa**	633	645	293	78 677
**3 ab**	635	645	244	52 083
**3 ac**	635	646	268	71 946
**3 ad**	632	641	222	66 051
**3 ae**	631	640	223	47 269
**3 af**	633	644	270	76 626
**3 ag**	633	643	246	76 107
**3 ah**	636	646	243	25 878
**3 bd**	637	644	158	11 431
**3 ca**	604	613	270	92 095
**5 aa**	630	639	223	71 598
**5 ab**	631	642	272	84 835
**7**	680	694	296	53 634

To investigate further the electronic structure of the DPPs **3**, we performed computational DFT studies for product **3 aa** at the B3LYP‐D3(BJ)/6–311+G(d,p)+SMD(*o*‐Xylene) level of theory (Figure 3).^25^ Our calculations showed that the HOMO is delocalized over the DPPs core and the ethylene bridge, whereas the LUMO is evenly localized on all condensed rings. Moreover, our TD‐DFT calculations, performed at the same level of theory, highlighted an optical gap (*E*_opt_) of 2.02 eV. Our computed absorption spectrum is in good qualitative agreement with the experimental data.^25^

**Figure 3:**
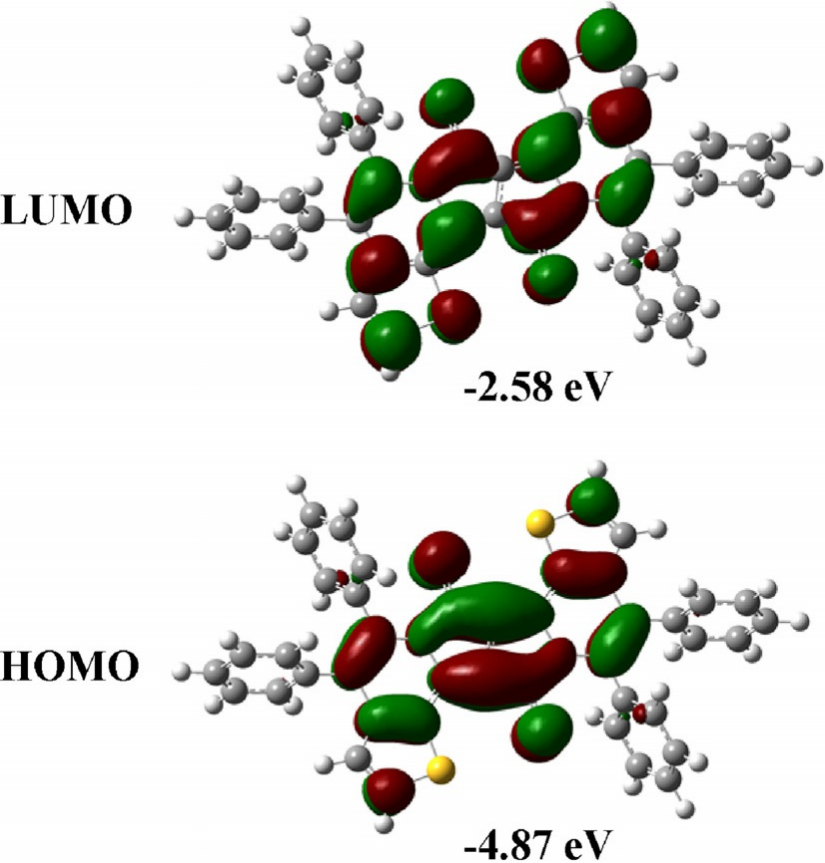
Energies and shapes of frontier orbitals (HOMO and LUMO) of **3 aa** calculated at the B3LYP‐D3(BJ)/6–311+G(d,p)+SMD(*o*‐Xylene) level of theory.

Finally, we became attracted by further late‐stage functionalization of PAH **3 am** in terms of the introduction of two aryl motifs17a at the alpha positions of the thiophenes (Scheme 4). Thus, the desired assembly of octylphenyl‐substituted DPP **7** was realized by palladium‐catalyzed twofold C−H arylations. It is noteworthy that the C−H activation‐based incorporation of two aromatic moieties drastically shifted both the absorption and the emission maxima into the NIR region, which was mirrored by the green color of PAH **7**.

**Scheme 4:**
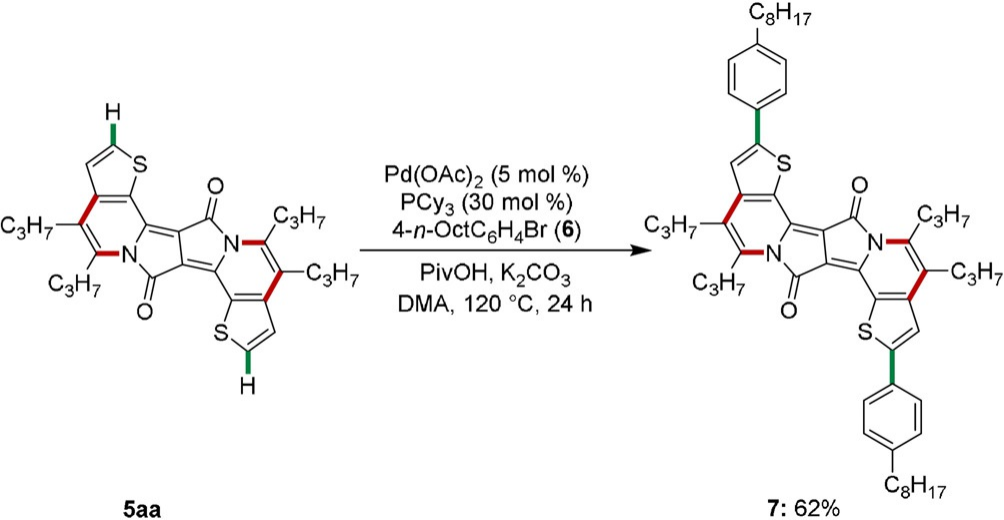
Late‐stage PAH diversification by double C−H arylations. Cy=cyclohexyl; DMA=dimethylacetamide; PivOH=pivalic acid.

In summary, we have devised an enabling strategy for the assembly of π‐extended DPPs. Thus, ruthenium(II)‐catalyzed double C−H/N−H activation allowed for the synthesis of novel, diversely‐decorated DPP derivatives in a step‐economical manner. The novel DPPs were fully characterized, including spectroscopy, XRD and DFT computation. The twofold alkyne annulation was furthermore merged with C−H arylations of the thus obtained π‐extended PAHs to furnish DPPs with absorption and emission maxima shifted into the NIR region. Our findings should prove invaluable for applications to optoelectronics, material sciences and live cell imaging.^28^

## Conflict of interest

The authors declare no conflict of interest.

## Supporting information

As a service to our authors and readers, this journal provides supporting information supplied by the authors. Such materials are peer reviewed and may be re‐organized for online delivery, but are not copy‐edited or typeset. Technical support issues arising from supporting information (other than missing files) should be addressed to the authors.

Supplementary

## References

[1] D. G. Farnum , G. Mehta , G. G. I. Moore , F. P. Siegal , Tetrahedron Lett. 1974, 15, 2549–2552.

[2a] C. Zhao , Y. Guo , Y. Zhang , N. Yan , S. You , W. Li , J. Mater. Chem. A 2019, 7, 10174–10199;

[2b] Y. Patil , R. Misra , Chem. Asian J. 2018, 13, 220–229; 29219247 10.1002/asia.201701493

[2c] W. Li , K. H. Hendriks , M. M. Wienk , R. A. J. Janssen , Acc. Chem. Res. 2016, 49, 78–85; 26693798 10.1021/acs.accounts.5b00334

[2d] M. J. Robb , S.-Y. Ku , F. G. Brunetti , C. J. Hawker , J. Polym. Sci. Part A 2013, 51, 1263–1271;

[2e] Y. Wu , W. Zhu , Chem. Soc. Rev. 2013, 42, 2039–2058; 23192709 10.1039/c2cs35346f

[2f] Y. Li , P. Sonar , L. Murphy , W. Hong , Energy Environ. Sci. 2013, 6, 1684–1710;

[2g] B. Tieke , A. R. Rabindranath , K. Zhang , Y. Zhu , Beilstein J. Org. Chem. 2010, 6, 830–845. 20978619 10.3762/bjoc.6.92 PMC2956471

[3] M. Kaur , D. H. Choi , Chem. Soc. Rev. 2015, 44, 58–77. 25186723 10.1039/c4cs00248b

[4a] M. Casutt , B. Dittmar , H. Makowska , T. Marszalek , S. Kushida , U. H. F. Bunz , J. Freudenberg , D. Jänsch , K. Müllen , Chem. Eur. J. 2019, 25, 2723–2728; 30624815 10.1002/chem.201806121

[4b] M. Grzybowski , D. T. Gryko , Adv. Opt. Mater. 2015, 3, 280–320;

[4c] O. Wallquist , R. Lenz , in High Performance Pigments (Eds.: E. B. Faulkner, R. J. Schwartz), 2009;

[4d] Z. Hao , A. Iqbal , Chem. Soc. Rev. 1997, 26, 203–213.

[5a] X. Zhang , R. Jin , Front. Chem. 2019, 7, 122; 30941343 10.3389/fchem.2019.00122 PMC6433785

[5b] Z. Liu , G. Zhang , D. Zhang , Acc. Chem. Res. 2018, 51, 1422–1432; 29771491 10.1021/acs.accounts.8b00069

[5c] S. D. Dimitrov , J. R. Durrant , Chem. Mater. 2014, 26, 616–630;

[5d] M. A. Naik , S. Patil , J. Polym. Sci. Part A 2013, 51, 4241–4260;

[5e] D. Gendron , M. Leclerc , Energy Environ. Sci. 2011, 4, 1225–1237.

[6a] M. Gsänger , D. Bialas , L. Huang , M. Stolte , F. Wurthner , Adv. Mater. 2016, 28, 3615–3645; 27028553 10.1002/adma.201505440

[6b] S. Holliday , J. E. Donaghey , I. McCulloch , Chem. Mater. 2014, 26, 647–663;

[6c] C. B. Nielsen , M. Turbiez , I. McCulloch , Adv. Mater. 2013, 25, 1859–1880. 23008141 10.1002/adma.201201795

[7] Y. Lin , Y. Li , X. Zhan , Chem. Soc. Rev. 2012, 41, 4245–4272. 22453295 10.1039/c2cs15313k

[8a] J. Wang , L. Liu , W. Xu , Z. Yang , Y. Yan , X. Xie , Y. Wang , T. Yi , C. Wang , J. Hua , Anal. Chem. 2019, 91, 5786–5793; 30938143 10.1021/acs.analchem.9b00014

[8b] S. Griesbeck , M. Evripidis , C. Wang , H. Ogasawara , S. Lorenzen , L. Gerstner , T. Zang , J. Nitsch , Y. Sato , R. Bertermann , M. Taki , C. Lambert , S. Yamaguchi , T. B. Marder , Chem. Sci. 2019, 10, 5405–5422. 31217943 10.1039/c9sc00793h PMC6549598

[9] X. Zhang , R. Sun , S. Sun , F. Ren , X. Chen , L. Wu , R. Xing , ACS Omega 2019, 4, 6068–6076. 31459754 10.1021/acsomega.9b00379 PMC6648016

[10] X. Yang , Q. Yu , N. Yang , L. Xue , J. Shao , B. Li , J. Shao , X. Dong , J. Mater. Chem. B 2019, 7, 2454–2462. 10.1039/c8tb03185a 32255122

[11] Y. Xu , J. Chen , L. Tong , P. Su , Y. Liu , B. Gu , B. Bao , L. Wang , J. Controlled Release 2019, 293, 94–103. 10.1016/j.jconrel.2018.11.016 30448086

[12] A. B. Pun , L. M. Campos , D. N. Congreve , J. Am. Chem. Soc. 2019, 141, 3777–3781. 30793886 10.1021/jacs.8b11796

[13] B. Soberats , M. Hecht , F. Würthner , Angew. Chem. Int. Ed. 2017, 56, 10771–10774; 10.1002/anie.201705137 28683178

[14] E. Heyer , P. Lory , J. Leprince , M. Moreau , A. Romieu , M. Guardigli , A. Roda , R. Ziessel , Angew. Chem. Int. Ed. 2015, 54, 2995–2999; 10.1002/anie.201411274 25630532

[15] S. Ghosh , R. Raveendran , A. Saeki , S. Seki , M. Namboothiry , A. Ajayaghosh , ACS Appl. Mater. Interfaces 2019, 11, 1088–1095. 30543390 10.1021/acsami.8b16714

[16] B. Carsten , F. He , H. J. Son , T. Xu , L. Yu , Chem. Rev. 2011, 111, 1493–1528. 21314190 10.1021/cr100320w

[17a] Z. Ni , H. Wang , H. Dong , Y. Dang , Q. Zhao , X. Zhang , W. Hu , Nat. Chem. 2019, 11, 271–277; 30692659 10.1038/s41557-018-0200-y

[17b] D. Feng , G. Barton , C. N. Scott , Org. Lett. 2019, 21, 1973–1978; 30860387 10.1021/acs.orglett.9b00019

[17c] S.-Y. Liu , D.-G. Wang , A.-G. Zhong , H.-R. Wen , Org. Chem. Front. 2018, 5, 653–661;

[17d] M. Wakiokam , F. Ozawa , Asian J. Org. Chem. 2018, 7, 1206–1216;

[17e] S. Yu , F. Liu , J. Yu , S. Zhang , C. Cabanetos , Y. Gao , W. Huang , J. Mater. Chem. C 2017, 5, 29–40.

[18a] T. Marks , E. Daltrozzo , A. Zumbusch , Chem. Eur. J. 2014, 20, 6494–6504; 24737556 10.1002/chem.201304235

[18b] G. M. Fischer , M. Isomäki-Krondahl , I. Göttker-Schnetmann , E. Daltrozzo , A. Zumbusch , Chem. Eur. J. 2009, 15, 4857–4864; 19296481 10.1002/chem.200801996

[18c] G. M. Fischer , A. P. Ehlers , A. Zumbusch , E. Daltrozzo , Angew. Chem. Int. Ed. 2007, 46, 3750–3753; 10.1002/anie.200604763 17410628

[19a] M. Hecht , B. Soberats , J. Zhu , V. Stepanenko , S. Agarwal , A. Greiner , F. Würthner , Nanoscale Horiz. 2019, 4, 169–174; 10.1039/c8nh00219c 32254152

[19b] T. He , P. Leowanawat , C. Burschka , V. Stepanenko , M. Stolte , F. Würthner , Adv. Mater. 2018, 30, 1804032; 10.1002/adma.201804032 30216567

[19c] W. Yue , S.-L. Suraru , D. Bialas , M. Müller , F. Würthner , Angew. Chem. Int. Ed. 2014, 53, 6159–6162; 10.1002/anie.201403227 24799332

[20a] M. Grzybowski , V. Hugues , M. Blanchard-Desce , D. T. Gryko , Chem. Eur. J. 2014, 20, 12493–12501; 25125043 10.1002/chem.201402569

[20b] M. Grzybowski , E. Glodkowska-Mrowka , T. Stoklosa , D. T. Gryko , Org. Lett. 2012, 14, 2670–2673. 22582959 10.1021/ol300674v

[21] F. Trilling , O. Sachnik , U. Scherf , Polym. Chem. 2019, 10, 627–632.

[22] For select review, see:

[22a] H. Ito , Y. Segawa , K. Murakami , K. Itami , J. Am. Chem. Soc. 2019, 141, 3–10; 30395456 10.1021/jacs.8b09232

[22b] P. Gandeepan , T. Müller , D. Zell , G. Cera , S. Warratz , L. Ackermann , Chem. Rev. 2019, 119, 2192–2452; 30480438 10.1021/acs.chemrev.8b00507

[22c] X. Liu , Y. Huang , X. Meng , J. Li , D. Wang , Y. Chen , D. Tang , B. Chen , Synlett 2019, 30, 207;

[22d] Y. Xiang , C. Wang , Q. Ding , Y. Peng , Adv. Synth. Catal. 2019, 361, 919–944;

[22e] Y. Koga , T. Kaneda , Y. Saito , K. Murakami , K. Itami , Science 2018, 359, 435–439; 29371465 10.1126/science.aap9801

[22f] S. Prakash , R. Kuppusamy , C. H. Cheng , ChemCatChem 2018, 10, 683–705;

[22g] S. J. Hein , D. Lehnherr , H. Arslan , F. J. Uribe-Romo , W. R. Dichtel , Acc. Chem. Res. 2017, 50, 2776–2788; 29112367 10.1021/acs.accounts.7b00385

[22h] W. Yang , W. A. Chalifoux , Synlett 2017, 28, 625–632;

[22i] R. Manikandan , M. Jeganmohan , Chem. Commun. 2017, 53, 8931–8947; 10.1039/c7cc03213g 28726865

[22j] M. Gulías , J. L. Mascareñas , Angew. Chem. Int. Ed. 2016, 55, 11000–11019; 10.1002/anie.201511567 27329931

[22k] Y. Segawa , T. Maekawa , K. Itami , Angew. Chem. Int. Ed. 2015, 54, 66–81; 10.1002/anie.201403729 25255741

[22l] L. Ackermann , Acc. Chem. Res. 2014, 47, 281–295; 23379589 10.1021/ar3002798

[22m] T. Jin , J. Zhao , N. Asao , Y. Yamamoto , Chem. Eur. J. 2014, 20, 3554–3576; 24591267 10.1002/chem.201304640

[22n] B. Li , P. H. Dixneuf , Chem. Soc. Rev. 2013, 42, 5744–5767; 23525331 10.1039/c3cs60020c

[22o] P. B. Arockiam , C. Bruneau , P. H. Dixneuf , Chem. Rev. 2012, 112, 5879–5918; 22934619 10.1021/cr300153j

[22p] L. Ackermann , R. Vicente , A. R. Kapdi , Angew. Chem. Int. Ed. 2009, 48, 9792–9826; 10.1002/anie.200902996 19998294

[22q] for select examples of simple C−H/N−H transformations, see: A. Obata , A. Sasagawa , K. Yamazaki , Y. Ano , N. Chatani , Chem. Sci. 2019, 10, 3242–3248; 30996908 10.1039/c8sc05063e PMC6430018

[22r] T. Wezeman , R. Scopelliti , F. F. Tirani , K. Severin , Adv. Synth. Catal. 2019, 361, 1383–1388;

[22s] M. Shankar , K. Ghosh , K. Mukherjee , R. K. Rit , A. K. Sahoo , Org. Lett. 2016, 18, 6416–6419; 27978646 10.1021/acs.orglett.6b03314

[22t] X. Zhang , W. Si , M. Bao , N. Asao , Y. Yamamoto , T. Jin , Org. Lett. 2014, 16, 4830–4833; 25203435 10.1021/ol502317c

[22u] B. Li , H. Feng , N. Wang , J. Ma , H. Song , S. Xu , B. Wang , Chem. Eur. J. 2012, 18, 12873–12879; 22930580 10.1002/chem.201201862

[22v] T. K. Hyster , T. Rovis , J. Am. Chem. Soc. 2010, 132, 10565–10569; 20662529 10.1021/ja103776u PMC2929375

[22w] K. Morimoto , K. Hirano , T. Satoh , M. Miura , Org. Lett. 2010, 12, 2068–2071; 20377234 10.1021/ol100560k

[22x] G. Song , D. Chen , C.-L. Pan , R. H. Crabtree , X. Li , J. Org. Chem. 2010, 75, 7487–7490; 20923219 10.1021/jo101596d

[22y] N. Umeda , H. Tsurugi , T. Satoh , M. Miura , Angew. Chem. Int. Ed. 2008, 47, 4019–4022; 10.1002/anie.200800924 18418815

[23a] L. Ackermann , Pure Appl. Chem. 2010, 82, 1403–1413;

[23b] L. Ackermann , Synlett 2007, 0507–0526.

[24] Selected examples:

[24a] Y. Qiu , C. Tian , L. Massignan , T. Rogge , L. Ackermann , Angew. Chem. Int. Ed. 2018, 57, 5818–5822; 10.1002/anie.201802748 29603565

[24b] R. Mei , J. Koeller , L. Ackermann , Chem. Commun. 2018, 54, 12879–12882; 10.1039/c8cc07732k 30376023

[24c] R. Mei , S.-K. Zhang , L. Ackermann , Synlett 2017, 28, 1715–1718;

[24d] L. Ackermann , L. Wang , A. V. Lygin , Chem. Sci. 2012, 3, 177–180;

[24e] L. Ackermann , A. V. Lygin , Org. Lett. 2012, 14, 764–767; 22242646 10.1021/ol203309y

[24f] L. Ackermann , A. V. Lygin , N. Hofmann , Org. Lett. 2011, 13, 3278–3281; 21612195 10.1021/ol201244s

[24g] L. Ackermann , A. V. Lygin , N. Hofmann , Angew. Chem. Int. Ed. 2011, 50, 6379–6382; 10.1002/anie.201101943 21612009

[25] For detailed information, see the Supporting Information.

[26] L. Ackermann , Chem. Rev. 2011, 111, 1315–1345. 21391562 10.1021/cr100412j

[27] C. Wang , H. Dong , L. Jiang , W. Hu , Chem. Soc. Rev. 2018, 47, 422–500. 29186226 10.1039/c7cs00490g

[28] Recent reviews:

[28a] C. Zhao , L. Mendive-Tapia , M. Vendrell , Arch. Biochem. Biophys. 2019, 661, 187–195; 30465736 10.1016/j.abb.2018.11.018

[28b] G. G. Dias , A. King , F. de Moliner , M. Vendrell , E. N. da Silva Júnior , Chem. Soc. Rev. 2018, 47, 12–27. 29099127 10.1039/c7cs00553a

