# Supplementary material for: π‐Extended Polyaromatic Hydrocarbons by Sustainable Alkyne Annulations through Double C−H/N−H Activation
Source: Chemistry. 2019 Dec 9;25(71):16246–50. doi: 10.1002/chem.201905023 (PMC6973059; doi:10.1002/chem.201905023)
Supplement: Supplementary file 1 — Supplementary [file CHEM-25-16246-s001.pdf]

CHEMISTRY
A European Journal

Supporting Information

π-Extended Polyaromatic Hydrocarbons by Sustainable Alkyne Annulations through Double C−H/N−H Activation

Elżbieta Gońka+, Long Yang+, Ralf Steinbock, Fabio Pesciaioli, Rositha Kuniyil, and Lutz Ackermann*[a]

chem_201905023_sm_miscellaneous_information.pdf

Table of Content

General Remarks S-3

Optimization..S-4

General Procedure For Double C–H/N–H Activation S-8

Characterization Data Of Products **3** and **5** S-9

Late-stage Functionalization of Compound **5aa** S-17

Variable Temperature NMR Studies S-18

X-Ray Crystallographic Analysis S-19

Computational Data...S-21

References S-27

NMR Spectra S-28

General Remarks

All catalytic reactions were carried out under a nitrogen atmosphere using pre-dried 25mL Schlenk tubes. Toluene, dimethylformamide (DMF), dichloromethane were purified by a MBraun MB SPS-800 solvent purification system. *tert*-Amyl alcohol (*t*-AmOH), *o*-Xylene were distilled over Na prior to its use. Dimethyl sulfoxide (DMSO), 1,2-dichloroethane (DCE) and dimethylacetamide (DMA) were distilled over CaH_2 prior to their use. The following starting materials were synthesized according to previously described methods: **1a**-**1c**,[1] **2b**-**2h**.[2] Acetylenes were distilled after purification by column chromatography. Other chemicals and solvents were obtained from commercial sources and used without further purification. Yields refer to isolated compounds, estimated to be >95% pure as determined by ^{1}H NMR. TLC: Merck, TLC Silica gel 60 F254. Chromatographic separations were carried out on Merck Silica 60 (0.040–0.063 mm). All IR spectra were recorded on a Bruker ATR FT-IR Alpha device. MS: EI-MS: JeolAccuTOF; ESI-MS: Bruker maXis and MicrOTOF. High resolution mass spectrometry (HRMS): Bruker maXis, Bruker MicrOTOF and Jeol AccuTOF. Melting points (M.p.): Stuart melting point apparatus SMP3, Barloworld Scientific, values are uncorrected. NMR spectra were recorded on Varian Mercury VX 300, Inova-500, Inova-600 and Bruker Avance 300, Avance III 300, Avance III HD 400, Avance III 400, Avance III HD 500 instruments, if not otherwise specified, chemical shifts (δ) are provided in ppm. UV-Vis absorption data were recorded on a Jasco® V-770 spectrophotometer. The scan speed was adjusted to 400 nm/min. Fluorescence excitation and emission data in solution were recorded on a Jasco® FP-8500 spectrofluorometer. The widths of excitation and emission slits were held constant at 5 and 5 nm, respectively. The scan speed was adjusted to 500 nm/min. The concentration in $CHCl_3$ is given for each sample and the excitation wavelengths were selected at the strongest signal.

Optimization

Optimization of Double C–H/N–H Activation of DPP With Aryl Alkyne 2a.[a]

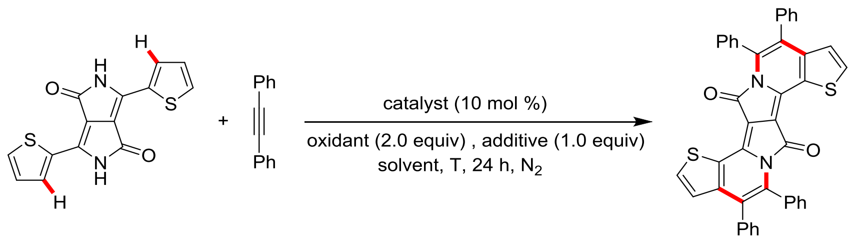

Entry	Catalyst	Oxidant	Additive	Solvent	T [°C]	Yield [%]
1	$Cp^*Co(CO)I_2$	$Cu(OAc)_2{\cdot}H_2O$	$AgSbF_6$	TFE	RT	--- [b]
2	$Cp^*Co(CO)I_2$	$Cu(OAc)_2{\cdot}H_2O$	$AgSbF_6$	TFE	100	--- [b]
3	[RuCl2(*p*-cymene)]2	$Cu(OAc)_2{\cdot}H_2O$	---	*t*-AmOH	100	--- [c]
4	[RuCl2(*p*-cymene)]2	$Cu(OAc)_2{\cdot}H_2O$	---	*t*-AmOH	100	< 5% [d]
5	[RuCl2(*p*-cymene)]2	$Cu(OAc)_2{\cdot}H_2O$	K_2CO_3	*t*-AmOH	100	--- [e]
6	[RuCl2(*p*-cymene)]2	$Cu(OAc)_2{\cdot}H_2O$	KOAc	*t*-AmOH	110	--- [f]
7	[RuCl2(*p*-cymene)]2	$Cu(OAc)_2{\cdot}H_2O$	KOAc	*t*-AmOH	100	< 5% [g]
8	[RuCl2(*p*-cymene)]2	$Cu(OAc)_2{\cdot}H_2O$	KOAc	*t*-AmOH	110	< 5% [g]
9	[RuCl2(*p*-cymene)]2	$Cu(OAc)_2{\cdot}H_2O$	---	*t*-AmOH	110	15%
10	[RuCl2(*p*-cymene)]2	$Cu(OAc)_2{\cdot}H_2O$	KOAc	*t*-AmOH	110	32% [h]
11	[RuCl2(*p*-cymene)]2	$CuBr_2$	---	*t*-AmOH	100	< 5% [c]
12	[RuCl2(*p*-cymene)]2	$CuBr_2$	KPF_6	*t*-AmOH	100	< 5% [f]
13	[RuCl2(*p*-cymene)]2	$CuBr_2$	NaOAc	*t*-AmOH	100	--- [f]
14	[RuCl2(*p*-cymene)]2	$CuBr_2$	NaOAc	*t*-AmOH	110	< 5% [g]
15	[RuCl2(*p*-cymene)]2	$CuBr_2$	KOAc	*t*-AmOH	100	--- [g]
16	[RuCl2(*p*-cymene)]2	$CuBr_2$	KOAc	*t*-AmOH	100	--- [f]
17	[RuCl2(*p*-cymene)]2	$CuBr_2$	KOAc	*t*-AmOH	110	< 5% [f]
18	[RuCl2(*p*-cymene)]2	$Cu(OAc)_2{\cdot}H_2O$	K_2CO_3	*t*-AmOH : DCE (1:1)	100	--- [e]
19	[RuCl2(*p*-cymene)]2	$Cu(OAc)_2{\cdot}H_2O$	K_2CO_3	*t*-AmOH : DCE (1:1)	100	--- [i]
20	[RuCl2(*p*-cymene)]2	$CuBr_2$	NaOAc	*t*-AmOH : DCE (1:1)	100	--- [f]
21	[RuCl2(*p*-cymene)]2	$Cu(OAc)_2{\cdot}H_2O$	KOAc	MTBE	70	--- [f]
22	[RuCl2(*p*-cymene)]2	$Cu(OAc)_2{\cdot}H_2O$	KOAc	MTBE	100	< 5% [g]
23	[RuCl2(*p*-cymene)]2	$Cu(OAc)_2{\cdot}H_2O$	---	MTBE	100	< 5% [d]
24	[RuCl2(*p*-cymene)]2	$Cu(OAc)_2{\cdot}H_2O$	KOAc	toluene	100	< 5% [f]
25	[RuCl2(*p*-cymene)]2	$Cu(OAc)_2{\cdot}H_2O$	---	*o*-xylene	140	35%
26	[RuCl2(*p*-cymene)]2	$Cu(OAc)_2{\cdot}H_2O$	---	*o*-xylene	140	22% [j]
27	[RuCl2(*p*-cymene)]2	$Cu(OAc)_2{\cdot}H_2O$	---	*o*-xylene	140	10% [k]
28	[RuCl2(*p*-cymene)]2	$Cu(OAc)_2{\cdot}H_2O$	---	*o*-xylene	140	25% [l]

29	$[RuCl_2(p\text{-cymene})]_2$	$Cu(OAc)_2·H_2O$	---	*o*-xylene	140	23% [m]
30	$[RuCl_2(p\text{-cymene})]_2$	$Cu(OAc)_2·H_2O$	---	*o*-xylene	140	68% [n]
31	$[RuCl_2(p\text{-cymene})]_2$	$Cu(OAc)_2·H_2O$	---	*o*-xylene	140	60% [o]
32	$[RuCl_2(p\text{-cymene})]_2$	$Cu(OAc)_2·H_2O$	---	*o*-xylene	140	64% [p]
33	$[RuCl_2(p\text{-cymene})]_2$	$Cu(OAc)_2·H_2O$	---	*o*-xylene	140	23% [q]
34	---	$Cu(OAc)_2·H_2O$	---	*o*-xylene	140	---
35	$[RuCl_2(p\text{-cymene})]_2$	$Cu(OAc)_2·H_2O$	KOAc [j]	*o*-xylene	140	30% [h]
36	$[RuCl_2(p\text{-cymene})]_2$	AgOAc	KOAc [j]	*o*-xylene	140	28% [h]
37	$[RuCl_2(p\text{-cymene})]_2$	$Cu(OAc)_2·H_2O$	KOAc	*o*-xylene	140	80%
38	$[RuCl_2(p\text{-cymene})]_2$	$Cu(OAc)_2·H_2O$	KOAc	*o*-xylene	140	43% [r]
39	$[RuCl_2(p\text{-cymene})]_2$	$Cu(OAc)_2·H_2O$	KOAc	*o*-xylene	140	69% [p]
40	$[RuCl_2(p\text{-cymene})]_2$ [n]	$Cu(OAc)_2·H_2O$	KOAc	*o*-xylene	140	15% [m]
41	$[RuCl_2(p\text{-cymene})]_2$	$Cu(OAc)_2·H_2O$	KOAc	DCE	140	70%
42	$[RuCl_2(p\text{-cymene})]_2$	$Cu(OAc)_2·H_2O$	KOAc	Toluene	140	56%
43	$[RuCl_2(p\text{-cymene})]_2$	$Cu(OAc)_2·H_2O$	KOAc	DMF	140	---
44	$[RuCl_2(p\text{-cymene})]_2$	$Cu(OAc)_2·H_2O$	KOAc	GVL	140	---
45	$[RuCl_2(p\text{-cymene})]_2$	$Cu(OAc)_2·H_2O$	KOAc	*o*-xylene	140	80% [s]
46	$[RuCl_2(p\text{-cymene})]_2$	$Cu(OAc)_2·H_2O$	KOAc	*o*-xylene	100	84%
47	$[RuCl_2(p\text{-cymene})]_2$	$Cu(OAc)_2·H_2O$	KOAc	*o*-xylene	100	92% [s]
48	$Pd(OAc)_2$	$Cu(OAc)_2·H_2O$	KOAc	*o*-xylene	140	--- [t]
49	$[Cp^*IrCl_2]_2$	$Cu(OAc)_2·H_2O$	KOAc	*o*-xylene	140	---
50	$[RhCp^*Cl_2]_2$	$Cu(OAc)_2·H_2O$	KOAc	*o*-xylene	140	76%
51	$[Ru(O_2CMes)_2(p\text{-cymene})]$	$Cu(OAc)_2·H_2O$	KOAc	*o*-xylene	140	66%[t]
52	---	$Cu(OAc)_2·H_2O$	KOAc	*o*-xylene	140	---
53	$[RhCp^*Cl_2]_2$	$Cu(OAc)_2·H_2O$	KOAc	*o*-xylene	100	20% [s]
54	$[RuCl_2(p\text{-cymene})]_2$	$CuBr_2$	KOAc	*o*-xylene	140	---
55	$[RuCl_2(p\text{-cymene})]_2$	$CuBr_2$	---	*o*-xylene	140	---

[a] Reaction conditions: **1a** (0.25 mmol), **2a** (1.0 mmol), catalyst (10 mol %), oxidant (2.0 equiv), additive (1.0 equiv), 2.0 mL of solvent, T, 24 h, N_2, isolated yields; [b] additive (20 mol %), 14 h; [c] oxidant (20 mol %), 20 h, under air; [d] oxidant (20 mol %), 20 h; [e] additive (1.5 equiv), 20 h, under air; [f] oxidant (20 mol %), additive (40 mol %), 20 h, under air; [g] oxidant (20 mol %), additive (40 mol %), 20 h; [h] additive (40 mol %); [i] oxidant (4.0 equiv), additive (1.5 equiv), 20 h, under air; [j] **2a** (0.5 mmol); [k] oxidant (20 mol %); [l] oxidant (4.0 equiv); [m] catalyst (5.0 mol %); [n] 48 h; [o] 96 h; [p] 3.0 mL of solvent; [q] catalyst (5.0 mol %), 3.0 mL of solvent; [r] set up in the glovebox; [s] 16 h; [t] catalyst (20 mol %).

Optimization of Ruthenium Catalyzed C–H/N–H Annulation of DPP With Alkyl Alkyne 4a.[a]

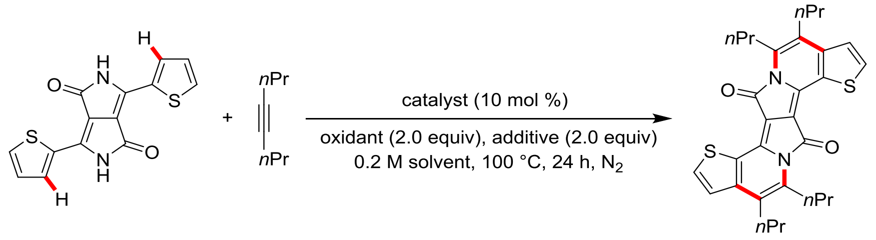

Entry	Catalyst	Oxidant	Additive	Solvent	T [°C]	Yield [%]
1	$[RuCl_2(p\text{-cymene})]_2$	$Cu(OAc)_2{\cdot}H_2O$	KOAc	*o*-xylene	100	25% [b]
2	$[RuCl_2(p\text{-cymene})]_2$	$Cu(OAc)_2{\cdot}H_2O$	KOAc	*o*-xylene	100	44% [c]
3	$[RuCl_2(p\text{-cymene})]_2$	$Cu(OAc)_2{\cdot}H_2O$	KPF_6	*o*-xylene	100	34% [d]
4	$[RuCl_2(p\text{-cymene})]_2$	$Cu(OAc)_2{\cdot}H_2O$	KOAc	*o*-xylene	130	35% [c]
5	$[RuCl_2(p\text{-cymene})]_2$	$Cu(OAc)_2{\cdot}H_2O$	KOAc	*o*-xylene	120	40%
6	$[RuCl_2(p\text{-cymene})]_2$	$Cu(OAc)_2{\cdot}H_2O$	KOAc	*o*-xylene	120	52% [e]
7	$[RuCl_2(p\text{-cymene})]_2$	$Cu(OAc)_2{\cdot}H_2O$	KOAc	DCM	50	--- [f]
8	$[RuCl_2(p\text{-cymene})]_2$	$Cu(OAc)_2{\cdot}H_2O$	Et_3N	*o*-xylene	100	<5%
9	$[RuCl_2(p\text{-cymene})]_2$	$Cu(OAc)_2{\cdot}H_2O$	HOAc	*o*-xylene	100	<5%
10	$[RuCl_2(p\text{-cymene})]_2$	AgOAc	KOAc	*o*-xylene	100	<5% [g]
11	$[Ru(OAc)_2(p\text{-cymene})]_2$	$Cu(OAc)_2{\cdot}H_2O$	KOAc	*o*-xylene	130	15%
12	$Cp^*Co(CO)I_2$	AgOAc	KOAc	*o*-xylene	100	<5% [g]
13	$[RhCp^*Cl_2]_2$	$Cu(OAc)_2{\cdot}H_2O$	KOAc	*o*-xylene	100	--- [h]
14	$[RuCl_2(p\text{-cymene})]_2$	$Cu(OAc)_2{\cdot}H_2O$	KOAc	*o*-xylene	100	43% [i]
15	$[RuCl_2(p\text{-cymene})]_2$	$Cu(OAc)_2{\cdot}H_2O$	KOAc	*o*-xylene	100	46%
16	$[RuCl_2(p\text{-cymene})]_2$	$Cu(OAc)_2{\cdot}H_2O$	KOAc	*o*-xylene	100	52% [j]
17	$[RuCl_2(p\text{-cymene})]_2$	$Cu(OAc)_2{\cdot}H_2O$	KOAc	*o*-xylene	100	25% [k]
18	$[RuCl_2(p\text{-cymene})]_2$	$Cu(OAc)_2{\cdot}H_2O$	KOAc	*o*-xylene	100	58% [f]
19	$[RuCl_2(p\text{-cymene})]_2$	$Cu(OAc)_2{\cdot}H_2O$	KOAc	*o*-xylene	100	58%
20	$[RuCl_2(p\text{-cymene})]_2$	$Cu(OAc)_2{\cdot}H_2O$	KOAc	*o*-xylene	100	53% [m]
21	$[RuCl_2(p\text{-cymene})]_2$	$Cu(OAc)_2{\cdot}H_2O$	KOAc	*o*-xylene	100	42% [n]
22	$[RuCl_2(p\text{-cymene})]_2$	$Cu(OAc)_2{\cdot}H_2O$	KOAc	*o*-xylene: DMSO (1:9)	100	<5%
23	$[RuCl_2(p\text{-cymene})]_2$	$Cu(OAc)_2{\cdot}H_2O$	KOAc	2-MeTHF	100	50% [f]
24	$[RuCl_2(p\text{-cymene})]_2$	$Cu(OAc)_2{\cdot}H_2O$	KOAc	2-MeTHF	100	42% [o]
25	$[RuCl_2(p\text{-cymene})]_2$	$Cu(OAc)_2{\cdot}H_2O$	KOAc	CF_3Ph	100	49% [f]

[a] Reaction conditions: **1a** (0.20 mmol), **4a** (0.80 mmol), catalyst (10 mol %), oxidant (4.0 equiv), additive (1.0 equiv), 1.0 mL of solvent, T, 24 h, N_2, isolated yields; [b] **4a** (0.40 mmol), oxidant (2.0 equiv.); [c] **4a** (0.40 mmol); [d] **4a** (0.40 mmol), additive (40 mol%); [e] work up w/o chromatography; [f] 16 h; [g] oxidant (3.0 equiv); [h] catalyst (5.0 mol %); [i] work up w/o chromatography, additive (50 mol%); [j] 2.0 mL of solvent; [k] catalyst (5.0

mol %), 2.0 mL of solvent; [l] oxidant (2.0 equiv), 3.0 mL of solvent; [m] 8 h; [n] oxidant (3.0 equiv), 2.0 mL of solvent; [o] 16 h, 2.0 mL of solvent.

General Procedure For Double C–H/N–H Activation:

General Procedure A for Double C–H/N–H Activation:

To the pre-oven dried 25 mL Schlenk flask diketopyrrolopyrrole **1** (0.25 mmol, 1.0 equiv), alkyne **2** (1.00 mmol, 4.0 equiv), $[RuCl_2(p\text{-cymene})]_2$ (10 mol %), $Cu(OAc)_2 \cdot H_2O$ (1.00 mmol, 4.0 equiv), KOAc (0.25 mmol, 1.0 equiv) were added. The Schlenk tube was evaporated and refilled with N_2, and *o*-xylene (2.0 mL) was added. The reaction was stirred at 100 °C for 16 h. At ambient temperature, the reaction mixture was transferred to a separation funnel with CH_2Cl_2 (3 x 500 mL) and washed with the saturated aqueous NH_4Cl and dried over Na_2SO_4. The solvent was removed under *vacuo* and the remaining residue was washed with *n*-hexane. The precipitate was separated with a centrifuge, dissolved in CH_2Cl_2 (1.0 L) and filtrated through filtration paper giving the desired product.

General Procedure B for Double C–H/N–H Activation:

To the pre-oven dried 25 mL Schlenk flask diketopyrrolopyrrole **1** (0.25 mmol, 1.0 equiv), alkyne **2** (1.00 mmol, 4.0 equiv), $[RuCl_2(p\text{-cymene})]_2$ (10 mol %), $Cu(OAc)_2 \cdot H_2O$ (1.00 mmol, 4.0 equiv), KOAc (0.25 mmol, 1.0 equiv) were added. The Schlenk tube was evaporated and refilled with N_2, and *o*-xylene (2.0 mL) was added. The reaction was stirred at 100 °C for 16 h. At ambient temperature, the reaction mixture was transferred to a separation funnel with CH_2Cl_2 (3 x 500 mL) and washed with the saturated aqueous NH_4Cl and dried over Na_2SO_4. The solvent was removed under *vacuo* and the remaining residue was purified by column chromatography on silica gel (*n*-hxane:CH_2Cl_2) giving the desired product.

Characterization Data of Products 3 and 5.

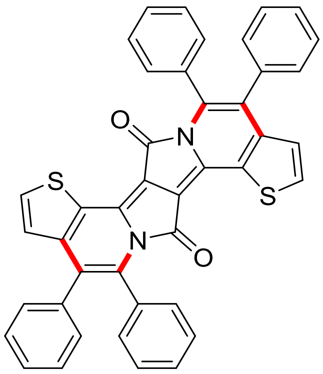

3aa

4,5,11,12-Tetraphenyl-7*H*,14*H*-thieno[3',2':7,8]indolizino[2,1-*a*]thieno[3,2-*g*]indolizine-7,14-dione (3aa):

The general procedure **A** was followed using 3,6-di(thiophen-2-yl)-2,5-dihydropyrrolo[3,4-c]pyrrole-1,4-dione (**1a**, 0.25 mmol, 75 mg) and 1,2-diphenylethyne (**2a**, 1.00 mmol, 178 mg) yielding **3aa** (149.8 mg, 92%) as purple crystals. M.p. > 360 °C. ^{1}H NMR (400 MHz, $CDCl_3$) δ = 7.59 (d, J = 5.2 Hz, 2H), 7.26 (m, 10H), 7.23 – 7.18 (m, 6H), 7.13–7.05 (m, 4H), 6.83 (dd, J = 5.1, 1.8 Hz, 2H). ^{13}C NMR (101 MHz, $CDCl_3$) δ = 156.5 (C_q), 143.5 (CH), 137.9 (CH), 136.7 (CH), 135.7 (CH), 133.3 (C_q), 132.6 (C_q), 131.6 (C_q), 130.6 (C_q), 130.3 (C_q), 128.3 (C_q), 128.0 (C_q), 127.2 (C_q), 124.7 (C_q), 123.8 (CH), 96.6 (CH). IR (ATR): 1683, 1633, 1480, 1346, 1015, 749, 694, 469 cm^{-1}. HR-MS (ESI) *m/z* calc. for $C_{42}H_{25}N_2O_2S_2^+$ $[M+H]^+$: 653.1352, found: 653.1356, *m/z* calcd. for $C_{42}H_{24}N_2NaO_2S_2^+$ $[M+Na]^+$: 675.1171, found: 675.1178.

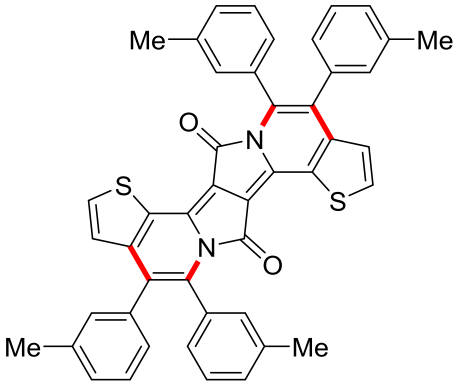

3ab

4,5,11,12-Tetra-*m*-tolyl-7*H*,14*H*-thieno[3',2':7,8]indolizino[2,1-*a*]thieno[3,2-*g*]indolizine-7,14-dione (3ab):

The general procedure **A** was followed using 3,6-di(thiophen-2-yl)-2,5-dihydropyrrolo[3,4-c]pyrrole-1,4-dione (**1a**, 0.2 mmol, 60 mg) and 1,2-di-*m*-tolylethyne (**2b**, 0.8 mmol, 165 mg)

yielding **3ab** (100 mg, 71%) as purple crystals. M.p. > 360 °C. ^{1}H NMR (300 MHz, $CDCl_3$) δ = 7.60 (d, J = 5.2 Hz, 2H), 7.20 – 6.80 (m, 18H), 2.35 – 2.15 (m, 12H). ^{13}C NMR (126 MHz, $CDCl_3$) δ = 156.5 (C_q), 143.6 (C_q), 138.0 (C_q), 137.4 (C_q), 137.3 (C_q), 136.7 (C_q), 136.4 (C_q), 136.3 (C_q), 135.7 (C_q), 133.1 (CH), 132.5 (C_q), 132.4 (C_q), 131.3 (CH), 131.2 (CH), 131.0 (CH), 130.9 (CH), 128.7 (CH), 128.7 (CH), 128.0 (C_q), 127.8 (CH), 127.7 (CH), 127.6 (CH), 127.6 (CH), 127.5 (CH), 127.4 (CH), 127.3 (CH), 126.8 (CH), 126.7 (CH), 124.8 (CH), 123.8 (C_q), 96.6 (C_q), 21.4 (CH_3), 21.3 (CH_3), 21.3 (CH_3). IR (ATR): 3089, 1687, 1628, 1502, 1347, 1095, 1033, 788, 757, 692, 490 cm^{-1}. HR-MS (ESI) m/z calc. for $C_{46}H_{33}N_2O_2S_2$ $[M+H]^+$: 709.1978, found: 709.1985.

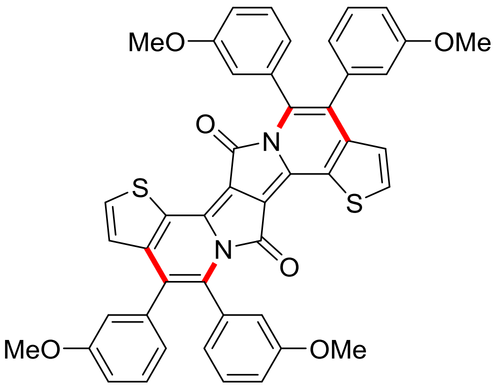

3ac

4,5,11,12-Tetrakis(3-methoxyphenyl)-7*H*,14*H*-thieno[3',2':7,8]indolizino[2,1-*a*]thieno[3,2-*g*]indolizine-7,14-dione (3ac):

The general procedure **A** was followed using 3,6-di(thiophen-2-yl)-2,5-dihydropyrrolo[3,4-c]pyrrole-1,4-dione (**1a**, 0.2 mmol, 60 mg) and 1,2-bis(3-methoxyphenyl)ethyne (**2c**, 0.8 mmol, 191 mg) yielding **3ac** (80 mg, 52%) as purple crystals. M.p. = 346 °C. ^{1}H NMR (400 MHz, $CDCl_3$) δ = 7.57 (d, J = 5.2 Hz, 2H), 7.22-7.07 (m, 4H), 6.98-6.93 (m, 1H), 6.89-6.83 (m, 3H), 6.83-6.60 (m, 9H), 6.51 (s, 1H), 3.70-3.59 (m, 12H). ^{13}C NMR (101 MHz, $CDCl_3$) δ = 159.1 (C_q), 159.1 (C_q), 158.5 (C_q), 158.4 (C_q), 156.3 (C_q), 143.3 (C_q), 137.5 (C_q), 137.0 (C_q), 137.0 (C_q), 136.7 (C_q), 133.7 (CH), 133.3 (CH), 129.0 (CH), 128.9 (CH), 128.2 (CH), 128.2 (CH), 128.0 (CH), 124.8 (CH), 123.5 (C_q), 123.1 (CH), 123.0 (CH), 122.9 (CH), 115.9 (CH), 115.9 (CH), 115.8 (CH), 115.7 (CH), 115.7 (CH), 114.0 (CH), 113.9 (CH), 113.5 (CH), 113.4 (CH), 113.2 (CH), 113.2 (CH), 96.6 (C_q), 55.2 (CH_3), 55.1 (CH_3), 55.1 (CH_3), 55.1 (CH_3). IR (ATR): 3090, 1685, 1632, 1575, 1475, 1284, 778, 756, 488 cm^{-1}. HR-MS (ESI) m/z calc. for $C_{46}H_{33}N_2O_6S_2^+$ $[M+H]^+$: 773.1775, found: 773.1766.

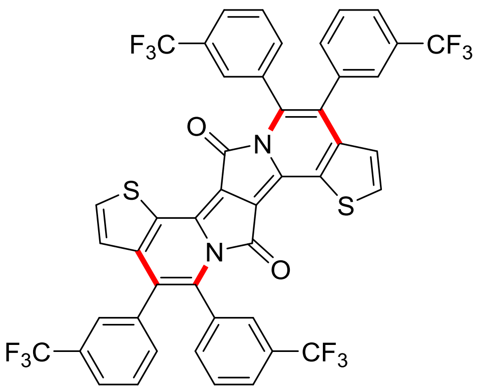

3ad

4,5,11,12-Tetrakis{3-(trifluoromethyl)phenyl}-7*H*,14*H*-thieno[3',2':7,8]indolizino[2,1-*a*]thieno[3,2-*g*]indolizine-7,14-dione (3ad):

The general procedure **A** was followed using 3,6-di(thiophen-2-yl)-2,5-dihydropyrrolo[3,4-c]pyrrole-1,4-dione (**1a**, 0.2 mmol, 60 mg) and 1,2-bis(3-methoxyphenyl)ethyne (**2d**, 0.8 mmol, 251 mg) yielding **3ad** (92 mg, 50%) as purple crystals. M.p. > 360 °C. ^{1}H NMR (400 MHz, $CDCl_3$) δ = 7.75 – 7.68 (m, 1H), 7.65 – 7.33 (m, 7H), 7.26 – 7.15 (m, 1H), 6.91 – 6.80 (m, 1H). ^{13}C NMR (126 MHz, $CDCl_3$) δ = 156.2 (C_q), 142.7 (C_q), 142.6 (C_q), 136.7 (C_q), 136.5 (C_q), 135.9 (C_q), 135.9 (C_q), 134.3 (CH), 134.3 (CH), 133.8 (CH), 133.5 (CH), 133.4 (CH), 133.4 (CH), 133.4 (CH), 133.3 (CH), 133.3 (CH), 133.3 (CH), 132.8 (C_q), 132.7 (C_q), 128.8 (CH), 128.7 (C_q), 127.7 (CH), 127.5 (CH), 127.3 (CH), 127.0 (CH), 125.1 (CH), 125.0 (CH), 124.8 (C_q), 124.8 (C_q), 124.8 (C_q), 124.8 (C_q), 124.7 (C_q), 124.4 (C_q), 124.4 (CH), 124.4 (CH), 124.3 (CH), 122.9 (C_q), 122.8 (C_q), 122.7 (C_q), 122.6 (C_q), 122.6 (C_q), 122.5 (C_q), 96.8 (C_q). ^{19}F NMR (471 MHz, $CDCl_3$): δ = -62.79, -62.93, -62.97, -63.08. IR (ATR): 2927, 2851, 1706, 1632, 1326, 1164, 1070, 803, 699, 664 cm^{-1}. HR-MS (ESI) m/z calc. for $C_{46}H_{21}F_{12}N_2O_2S_2^+$ [M+H]$^+$: 925.0847, found: 925.0839.

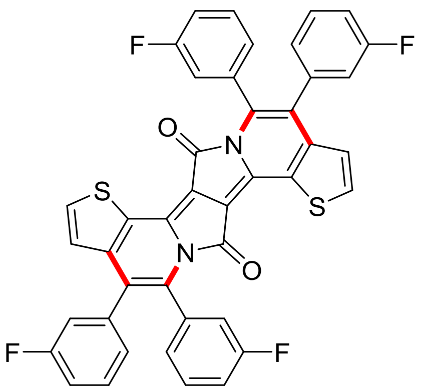

3ae

4,5,11,12-Tetrakis(3-fluorophenyl)-7*H*,14*H*-thieno[3',2':7,8]indolizino[2,1-*a*]thieno[3,2-*g*]indolizine-7,14-dione (3ae):

The general procedure **A** was followed using 3,6-di(thiophen-2-yl)-2,5-dihydropyrrolo[3,4-c]pyrrole-1,4-dione (**1a**, 0.2 mmol, 60 mg) and 1,2-bis(3-methoxyphenyl)ethyne (**2e**, 0.8 mmol, 171 mg) yielding **3ae** (90 mg, 62%) as purple crystals. M.p. > 360 °C. ^{1}H NMR (500 MHz, $CDCl_3$) δ = 7.62 (d, J = 5.2 Hz, 2H), 7.24–7.14 (m, 4H), 7.06 (d, J = 7.6 Hz, 2H), 7.03–6.74 (m, 12H). ^{13}C NMR (126 MHz, $CDCl_3$) δ = 162.4 (d, $^1J_{C\text{-}F}$ = 247 Hz, C_q), 161.8 (d, $^1J_{C\text{-}F}$ = 247 Hz, C_q), 156.2 (C_q), 142.9 (C_q), 137.3 (d, $^4J_{C\text{-}F}$ = 7.9 Hz, C_q), 136.6 (C_q), 136.4 (C_q), 134.1 (d, $^3J_{C\text{-}F}$ = 8.5 Hz, C_q), 133.9 (CH), 129.8 (d, $^3J_{C\text{-}F}$ = 9 Hz, CH), 129.7 (d, $^3J_{C\text{-}F}$ = 9 Hz, CH), 129.0 (d, $^3J_{C\text{-}F}$ = 8.5 Hz, CH), 128.8 (d, $^4J_{C\text{-}F}$ = 8.3 Hz, CH), 128.4 (C_q), 126.2 (d, $^2J_{C\text{-}F}$ = 19.5 Hz, CH), 124.5 (CH), 122.6 (C_q), 117.4 (d, $^2J_{C\text{-}F}$ = 20 Hz, CH), 115.4 (d, $^2J_{C\text{-}F}$ = 21 Hz, CH), 114.6 (d, $^2J_{C\text{-}F}$ = 21 Hz, CH), 96.7 (C_q). ^{19}F NMR (471 MHz, $CDCl_3$): δ = –112.58, -112.85, -113.30, -113.56. IR (ATR): 1702, 1610, 1582, 1479, 1431, 1146, 758, 695, 489 cm^{-1}. HR-MS (ESI) m/z calc. for $C_{42}H_{21}F_4N_2O_2S_2^+$ [M+H]$^+$: 725.0975, found: 725.0961.

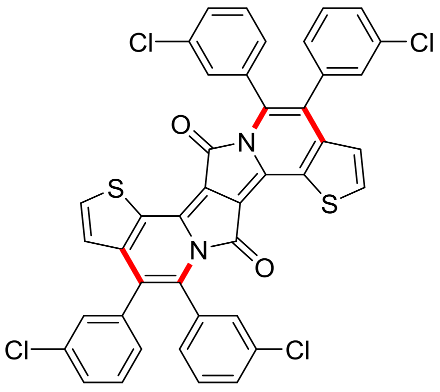

3af

4,5,11,12-Tetrakis(3-chlorophenyl)-7*H*,14*H*-thieno[3',2':7,8]indolizino[2,1-*a*]thieno[3,2-*g*]indolizine-7,14-dione (3af):

The general procedure **A** was followed using 3,6-bis(5-octylthiophen-2-yl)-2,5-dihydropyrrolo[3,4-*c*]pyrrole-1,4-dione (**1a**, 0.2 mmol, 60 mg) and 1,2-bis(3-chlorophenyl)ethyne (**2f**, 0.8 mmol, 198 mg) yielding **3af** (81 mg, 51%) as purple crystals M.p. > 360 °C. ^{1}H NMR (400 MHz, $CDCl_3$) δ = 7.68 (d, J = 5.1 Hz, 2H), 7.28 – 7.15 (m, 13H), 7.11 (s, 1H), 7.02 (s, 1H), 6.96 (d, J = 7.7 Hz, 1H), 6.91 – 6.83 (m, 2H). ^{13}C NMR (101 MHz, $CDCl_3$) 156.2 (C_q), 142.9 (C_q), 137.0 (C_q), 136.7 (C_q), 136.4 (C_q), 134.2 (C_q), 134.1 (C_q), 134.0 (CH), 133.9 (C_q), 133.5 (C_q), 133.3 (C_q), 130.5 (CH), 130.4 (CH), 130.2 (CH), 129.6 (CH), 129.5 (CH), 128.8 (CH), 128.7 (CH), 128.6 (CH), 128.5 (CH), 127.9 (CH), 124.5 (CH), 122.6 (C_q),

96.7 (C_q). IR (ATR): 1698, 1635, 1565, 1472, 1095, 1029, 789, 751, 707,486 cm^{-1}. HR-MS (ESI) m/z calc. for $C_{42}H_{21}{}^{35}Cl_4N_2O_2S_2^+$ $[M+H]^+$: 788.9793, found: 788.9787.

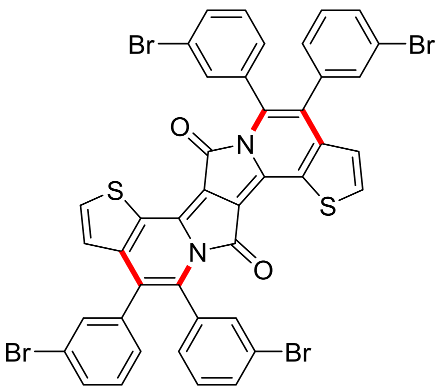

3ag

4,5,11,12-Tetrakis(3-bromophenyl)-7*H*,14*H*-thieno[3',2':7,8]indolizino[2,1-*a*]thieno[3,2-*g*]indolizine-7,14-dione (3ag):

The general procedure **A** was followed using 3,6-bis(5-octylthiophen-2-yl)-2,5-dihydropyrrolo[3,4-*c*]pyrrole-1,4-dione (**1a**, 0.2 mmol, 60 mg) and 1,2-bis(3-bromophenyl)ethyne (**2g**, 0.8 mmol, 269 mg) yielding **3ag** (103 mg, 53%) as purple crystals. M.p. > 360 °C. ^1H NMR (300 MHz, $CDCl_3$) δ = 7.68 (d, J = 5.2 Hz, 2H), 7.47 – 7.37 (m, 6H), 7.34 (s, 1H), 7.27 – 7.05 (m, 8H), 6.99 (d, J = 7.9 Hz, 1H), 6.87 (d, J = 5.4 Hz, 2H). ^{13}C NMR (126 MHz, $CDCl_3$) δ = 156.2 (C_q), 142.8 (C_q), 137.2 (C_q), 137.1 (C_q), 136.6 (C_q), 136.3 (C_q), 134.0 (CH), 133.5 (CH), 133.3 (CH), 133.0 (CH), 132.9 (CH), 131.4 (CH), 130.8 (CH), 129.8 (CH), 129.7 (CH), 129.2 (CH), 129.1 (CH), 128.9 (CH), 128.7 (CH), 128.5 (C_q), 124.5, 122.6 (C_q), 122.2 (C_q), 122.1 (C_q), 121.5 (C_q), 121.4 (C_q), 96.7 (C_q). IR (ATR): 1697, 1635, 1559, 1470, 1347, 1094, 787, 746, 682, 482 cm^{-1}. HR-MS (ESI) m/z calc. for $C_{42}H_{21}{}^{79}Br_4N_2O_2S_2^+$ $[M+H]^+$: 964.7772, found: 964.7712.

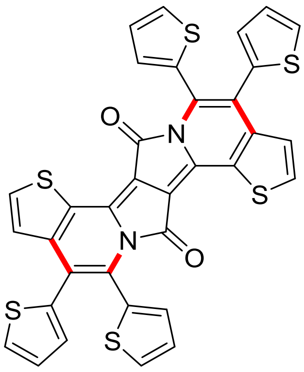

3ah

4,5,11,12-Tetra(thiophen-2-yl)-7*H*,14*H*-thieno[3',2':7,8]indolizino[2,1-*a*]thieno[3,2-*g*]indolizine-7,14-dione (3ah):

The general procedure **A** was followed using 3,6-di(thiophen-2-yl)-2,5-dihydropyrrolo[3,4-c]pyrrole-1,4-dione (**1a**, 0.25 mmol, 75 mg) and 1,2-di(thiophen-2-yl)ethyne (**2h**, 1.00 mmol, 190 mg) yielding **3ah** (135 mg, 79%) as blue powder. M.p. > 360 °C. ^{1}H NMR (300 MHz, $CDCl_3$) δ = 7.64 (d, J = 5.2 Hz, 2H), 7.43 (d, J = 5.2 Hz, 2H), 7.30 (m, 2H), 7.11 (d, J = 3.2 Hz, 2H), 7.05 (d, J = 5.2 Hz, 2H), 7.04 – 6.90 (m, 6H). ^{13}C NMR (126 MHz, $CDCl_3$) δ = 143.3 (C_q), 135.9 (C_q), 133.7 (C_q), 132.2 (C_q), 130.6 (CH), 129.1 (CH), 128.7 (C_q), 127.7 (C_q), 126.8 (CH), 126.5 (CH), 126.2 (CH), 125.0 (C_q), 119.2 (CH), 96.8 (C_q). IR (ATR): 1681, 1632, 1558, 1402, 1141, 1081, 1038, 693, 476 cm^{-1}. HR-MS (ESI): m/z calc. for $C_{34}H_{17}N_2O_2S_6^+$ [M+H]$^+$ 676.9614, found: 676.9609.

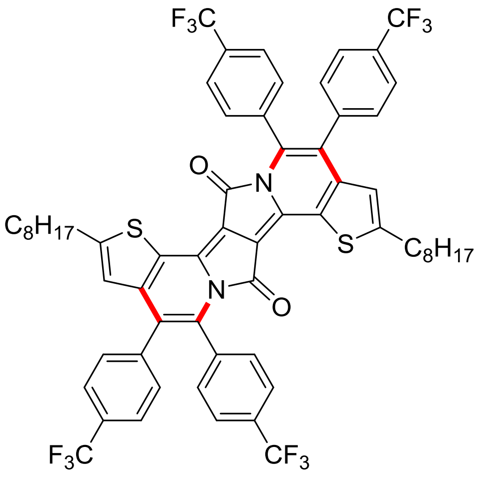

3bd

2,9-Di-*n*-octyl-4,5,11,12-tetrakis(4-(trifluoromethyl)phenyl)-7*H*,14*H*-thieno[3',2':7,8]indolizino[2,1-*a*]thieno[3,2-*g*]indolizine-7,14-dione (3bd):

The general procedure **A** was followed using 3,6-bis(5-octylthiophen-2-yl)-2,5-dihydropyrrolo[3,4-*c*]pyrrole-1,4-dione (**1b**, 0.25 mmol, 132 mg) and 1,2-bis(4-(trifluoromethyl)phenyl)ethyne (**2d**, 1.00 mmol, 314 mg) yielding **3bd** (136 mg, 47%) as a blue powder. M.p. > 360 °C. ^{1}H NMR (300 MHz, $CDCl_3$) δ = 7.50 (m, 4H), 7.38 (d, J = 8.0 Hz, 2H), 7.18 (d, J = 7.9 Hz, 2H), 6.42 (s, 1H), 2.76 (t, J = 7.7 Hz, 2H), 1.61 (d, J = 7.6 Hz, 2H), 1.22 (m, 10H), 0.85 (t, J = 6.5 Hz, 3H). ^{13}C NMR (101 MHz, $CDCl_3$) δ = 156.4 (C_q), 155.8 (CH), 142.6 (CH), 138.8 (CH), 136.1 (CH), 135.7 (CH), 130.5 (C_q), 130.3 (C_q), 129.9 (CH), 129.6 (CH), 129.2 (CH), 127.0 (CH), 124.9 (C_q), 124.8 (C_q), 124.8 (C_q), 124.0 (C_q), 124.0 (C_q), 122.2 (CH), 122.1 (CH), 122.0 (CH), 120.9 (C_q), 95.5 (CH_2), 31.4 (CH_2), 31.0 (CH_2), 30.7 (CH_2), 28.8 (CH_2), 28.7 (CH_2), 22.2 (CH_2), 13.7 (CH_3). ^{19}F NMR (377 MHz, $CDCl_3$) δ = –62.64, –

62.70. IR (ATR): 1699, 1616, 1553, 1323, 1260, 1067, 1017, 799 cm^{-1}. HR-MS (ESI) m/z calc. for $C_{62}H_{53}F_{12}N_2O_2S_2^+$ [M+H]$^+$: 1149.3357, found: 1149.3351.

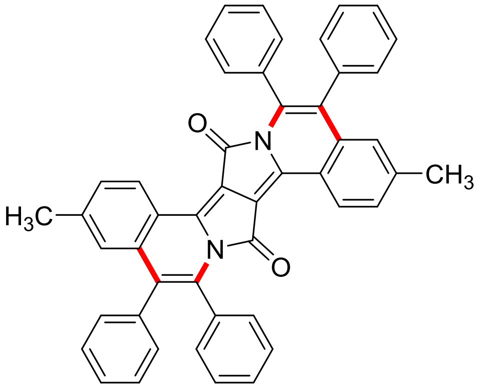

3ca

6,18-Dimethyl-9,10,21,22-tetraphenyl-11.23-diazahexacyclo[11.11.0.0^{2,11}.0^{3,8}.0^{14,23}.0^{15,20}]tetracosa-1,3(8),4,6,9,13,15(20),16,18,21-decaene-12,24-dione (3ca):

The general procedure **B** was followed using 3,6-di-p-tolyl-2,5-dihydropyrrolo[3,4-c]pyrrole-1,4-dione (**1c**, 0.25 mmol, 62 mg) and 1,2-diphenylethyne (**2a**, 1.00 mmol, 178 mg). Isolation by column chromatography (*n*-hexane/CH_2Cl_2 1/3) yielding **3ca** (70.3 mg, 42%) as pink crystals. M.p. > 360 °C. ^{1}H NMR (400 MHz, $CDCl_3$) δ = 9.22 (d, J = 8.3 Hz, 2H), 7.20-7.27 (m, 18H), 7.10 – 7.07 (m, 4H), 6.85 (s, 2H), 2.33 (s, 6H). ^{13}C NMR (101 MHz, $CDCl_3$) δ = 157.5 (C_q), 143.3 (C_q), 142.2 (C_q), 137.2 (C_q), 135.4 (C_q), 134.8 (C_q), 134.1 (C_q), 131.4 (CH), 129.9 (CH), 129.8 (CH), 129.5 (CH), 127.9 (CH), 127.6 (CH), 127.1 (CH), 127.0 (CH), 125.7 (CH), 125.5 (C_q), 122.2 (C_q), 101.1 (C_q), 22.3 (CH_3). IR (ATR): 1677, 1610, 1479, 1331, 1031, 774, 693, 438 cm^{-1}. HR-MS (EI) *m/z* calcd for $C_{48}H_{33}N_2O_2^+$ [M+H]$^+$: 669.2537, found: 669.2539.

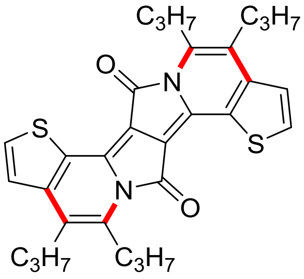

5aa

4,5,11,12-Tetrapropyl-7*H*,14*H*-thieno[3',2':7,8]indolizino[2,1-a]thieno[3,2-g]indolizine-7,14-dione (5aa):

The general procedure **A** was followed using 3,6-di(thiophen-2-yl)-2,5-dihydropyrrolo[3,4-c]pyrrole-1,4-dione (**1a**, 0.2 mmol, 60 mg) and oct-4-yne (**4a**, 0.8 mmol, 88 mg) yielding **5aa** (60 mg, 58%) as purple crystals. M.p. = 333 °C. ^{1}H NMR (300 MHz, $CDCl_3$) δ = 7.74 (d, J = 5.3 Hz, 2H), 7.27 (d, 2H), 3.48-3.39 (m, 4H), 2.80-2.71 (m, 4H), 1.86-1.76 (m, 4H), 1.72-1.62 (m, 4H), 1.14 (t, J = 6.6 Hz, 6H), 1.09 (t, J = 6.6 Hz, 6H). ^{13}C NMR (126 MHz, $CDCl_3$) δ = 157.5 (C_q), 143.5 (C_q), 141.1 (C_q), 137.2 (C_q), 133.1 (CH), 127.4 (C_q), 122.8 (CH), 120.2 (C_q), 96.1 (C_q), 30.9 (CH_2), 30.6 (CH_2), 28.2 (CH_2), 24.1 (CH_2), 23.7 (CH_2), 14.4 (CH_3), 14.3 (CH_3). IR (ATR): 3074, 2963, 2951, 2928, 2867, 1683, 1640, 1546, 1028, 768, 698, 467 cm^{-1}. HR-MS (ESI) m/z calc. for $C_{30}H_{33}N_2O_2S_2$ [M+H]$^+$: 517.1978, found: 517.1974.

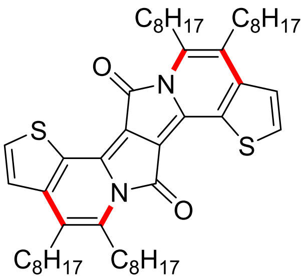

5ab

4,5,11,12-Tetrakis(3-octylphenyl)-7*H*,14*H*-thieno[3',2':7,8]indolizino[2,1-*a*]thieno[3,2-*g*]indolizine-7,14-dione (5ab):

The general procedure **A** was followed using 3,6-di(thiophen-2-yl)-2,5-dihydropyrrolo[3,4-c]pyrrole-1,4-dione (**1a**, 0.2 mmol, 60 mg) and octadec-9-yne (**4b**, 0.8 mmol, 200 mg) yielding **5ab** (89 mg, 56%) as purple crystals. M.p. = 287 °C. ^{1}H NMR (400 MHz, $CDCl_3$) δ = 7.73 (d, J = 5.2 Hz, 2H), 7.27 (d, J = 5.5 Hz, 2H), 3.44 (t, J = 8.0 Hz, 4H), 2.75 (t, J = 8.1 Hz, 4H), 1.81 – 1.71 (m, 4H), 1.68 – 1.59 (m, 4H), 1.53 – 1.28 (m, 40H), 0.95 – 0.88 (m, 12H). ^{13}C NMR (101 MHz, $CDCl_3$) δ = 157.5 (C_q), 143.6 (C_q), 141.3 (C_q), 137.1 (C_q), 133.1 (CH), 127.4 (C_q), 122.7 (CH), 120.3 (C_q), 96.1 (C_q), 31.9 (CH_2), 31.9 (CH_2), 30.8 (CH_2), 30.5 (CH_2), 29.9 (CH_2), 29.8 (CH_2), 29.5 (CH_2), 29.4 (CH_2), 29.3 (CH_2), 28.6 (CH_2), 26.3 (CH_2), 22.7 (CH_2), 22.7 (CH_2), 14.1 (CH_3), 14.1 (CH_3). IR (ATR): 2954, 2918, 2848, 1676, 1538, 1505, 1349, 1017, 772, 700 cm^{-1}. HR-MS (ESI) m/z calc. for $C_{50}H_{73}N_2O_2S_2$ [M+H]$^+$: 797.5108, found: 797.5091.

Late-stage Functionalization of Compound 5aa

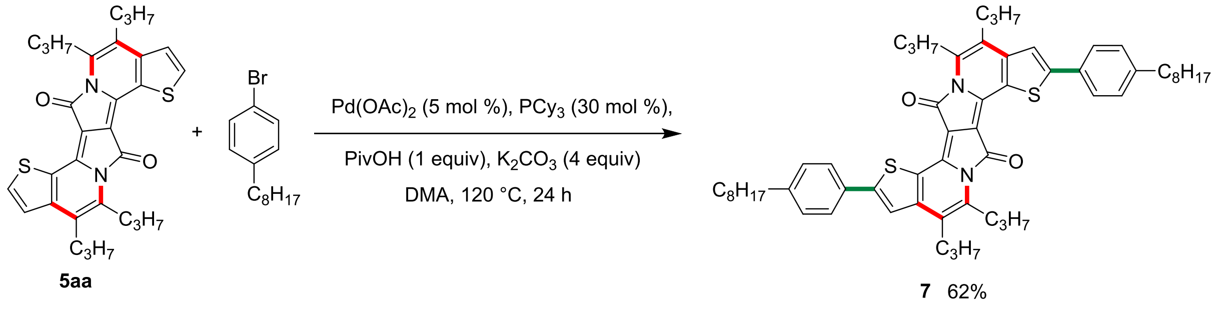

To an oven dried 25 mL Schlenk flask compound **5aa** (0.10 mmol, 51.6 mg) and $Pd(OAc)_2$ (10 mol %, 2.2 mg) were added. Under N_2, PCy_3 (30 mol %, 8.4 mg), PivOH (0.10 mmol, 10.2 mg) and K_2CO_3 (0.40 mmol, 55.3 mg) were added to the Schlenk flask inside the glovebox. Next DMA (1.0 mL), followed by 1-bromo-4-octylbenzene (0.20 mmol, 53.8 mg) were added. The reaction was stirred at 120 °C for 24 h. At ambient temperature, the reaction mixture was transferred to a separation funnel with CH_2Cl_2 (3 x 50 mL) washed with H_2O and dried over Na_2SO_4. The solvent was removed in vacuo and the remaining residue was purified by column chromatography on silica gel (*n*-hexane/CH_2Cl_2) to give product **7** (55 mg, 62%) as green crystals. M.p. = 247.2 °C. ^{1}H NMR (300 MHz, $CDCl_3$) δ = 7.72 (d, J = 7.9 Hz, 4H), 7.36 (s, 2H), 7.25 (d, J = 7.8 Hz, 4H), 3.43 (dd, J = 9.5, 6.4 Hz, 4H), 2.74 (t, J = 8.1 Hz, 4H), 2.65 (t, J = 7.7 Hz, 4H), 1.82 (m, 4H), 1.66 (m, 8H), 1.30 (d, J = 12.4 Hz, 20H), 1.12 (m, 12H), 0.97 – 0.80 (m, 6H). ^{13}C NMR (101 MHz, $CDCl_3$) δ = 157.7 (C_q), 151.8 (C_q), 144.7 (C_q), 144.6 (C_q), 141.5 (C_q), 136.4 (C_q), 130.9 (C_q), 129.2, 126.8, 120.1 (C_q), 117.7 (CH_2), 96.5 (C_q), 35.9 (CH_2), 32.0 (CH_2), 31.5 (CH_2), 30.8 (CH_2), 29.6 (CH_2), 29.5 (CH_2), 29.4 (CH_2), 28.3 (CH_2), 24.3 (CH_2), 23.8 (CH_2), 22.8 (CH_2), 14.6 (CH_3), 14.5 (CH_3), 14.2 (CH_3). IR (ATR): 2956, 2921, 2851, 1675, 1627, 1505, 1352, 806, 480 cm^{-1}. HR-MS (ESI) m/z calc. for $C_{58}H_{73}N_2O_2S_2^+$ [M+H]$^+$: 893.5108, found: 893.5110.

Variable Temperature NMR Studies

^{1}H NMR spectra for compound **3ab** was measured at different temperatures using D_2-1,1,2,2-tetrachloroethane as the solvent (Figure S-1). In comparison to the ^{1}H NMR spectrum measured at 298 K, at higher temperature (338 K) only two resonances are visible in alkyl range. Our observation clearly showed that the lack of symmetry, observed at room temperature of *meta*-substituted derivatives, is caused by the presence of rotameric isomers.

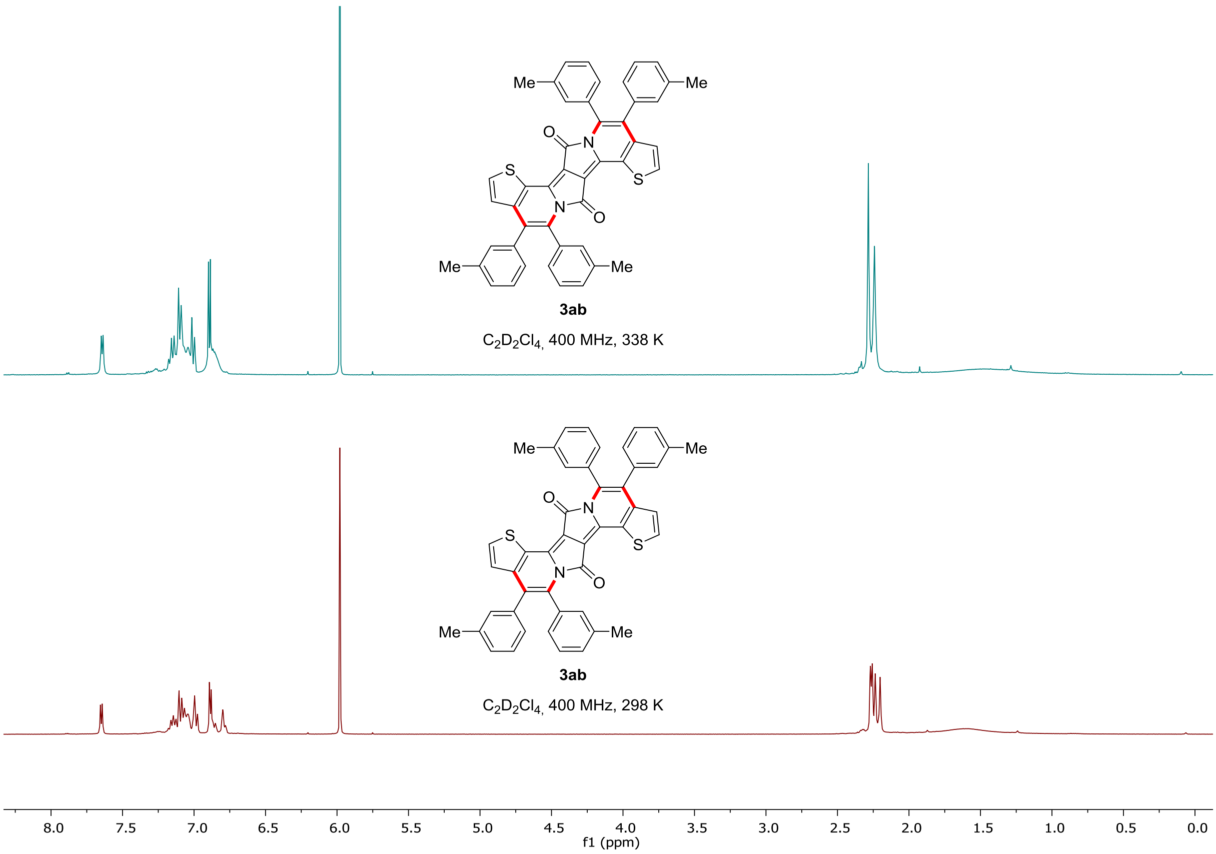

Figure S-1. 1H NMR spectra of compound **3ab** measured at 298 K and 338 K.

X-Ray Crystallographic Analysis

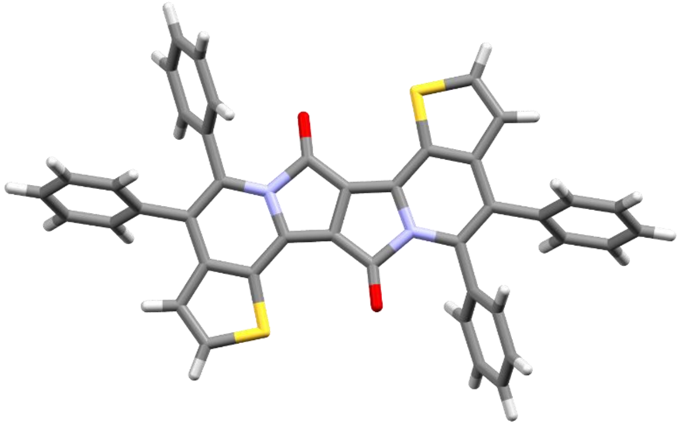

Crystal data and structure refinement for 0423_CG_0m.

Identification code	0423_CG_0m
Empirical formula	$C_{44}H_{26}Cl_6N_2O_2S_2$
Formula weight	891.49
Temperature/K	100.02
Crystal system	triclinic
Space group	P-1
a/Å	9.4892(16)
b/Å	9.9820(14)
c/Å	11.1219(17)
α/°	83.678(5)
β/°	77.410(6)
γ/°	70.646(5)
Volume/Å^3	969.2(3)
Z	1
ρ_{calc}g/cm^3	1.527
μ/mm^{-1}	0.594
F(000)	454.0
Crystal size/mm^3	0.216 × 0.088 × 0.048
Radiation	MoKα (λ = 0.71073)
2Θ range for data collection/°	4.638 to 61.144
Index ranges	-13 ≤ h ≤ 13, -14 ≤ k ≤ 14, -15 ≤ l ≤ 15
Reflections collected	31016
Independent reflections	5943 [R_{int} = 0.0245, R_{sigma} = 0.0190]
Data/restraints/parameters	5943/18/281
Goodness-of-fit on F^2	1.100

Final R indexes [I>=2σ (I)]	R_1 = 0.0378, wR_2 = 0.0963
Final R indexes [all data]	R_1 = 0.0412, wR_2 = 0.0985
Largest diff. peak/hole / e Å^{-3}	0.59/-0.32

Computational Data

All calculations were carried out by using DFT with the Gaussian 16, Revision A.03 package.[3] Geometry optimisation of the compound **3aa** was carried out at the B3LYP[4] level of theory in combination with D3 dispersion corrections with Becke-Johnson damping scheme (D3BJ).[5] The 6-311+G(d,p) basis set was used for all the atoms.[6] Analytical frequency calculations were carried out at the same level of theory in order to identify the intermediate (no imaginary frequency). Solvent effects were taken into consideration during the optimisation. These were implicitly included through the use of the SMD[7] model with a dielectric constant of ε = 2.5454, which corresponds to *o*-Xylene.

Time-dependent (TD)-DFT calculations were carried out on the optimized structure of 3xy using the same level of theory and basis set as for the geometry optimisation to obtain the absorption spectra.

Plots of frontier orbitals were constructed by using Gauss View 5 software.[8]

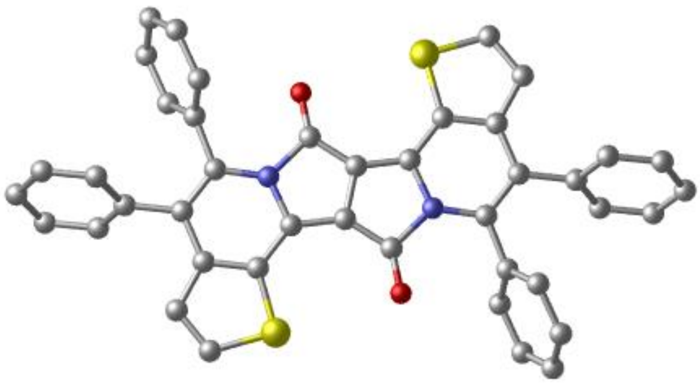

Figure S-2. Optimized geometry of compound **3aa** at the B3LYP D3(BJ)/6-311+G(d,p)+ SMD(o-Xylene) level of theory.

Table S-1. Absorption spectra data obtained from TD-DFT calculations for the compound 3xy at the B3LYP D3(BJ)/6-311+G(d,p)+SMD(o-Xylene) level of theory.

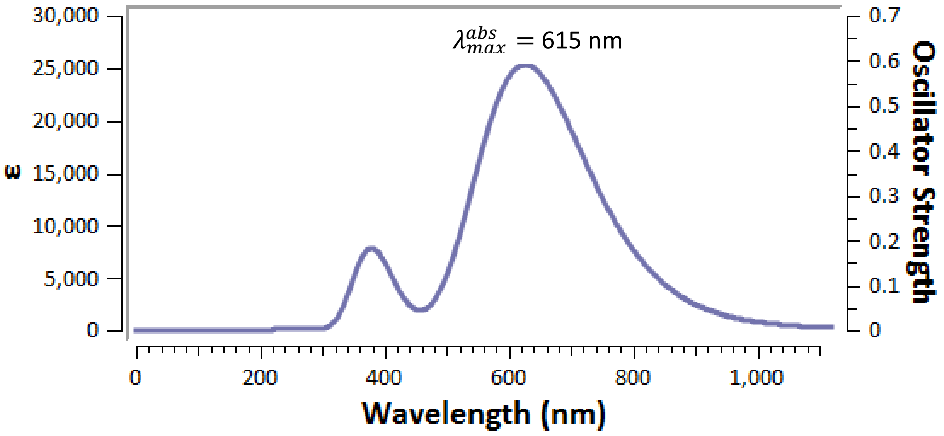

λ_{max} (nm)	Oscillator Strength (*f*)	Excitation	Coefficient
615.44 nm	0.6233	169 → 170	0.70597
467.92 nm	0.0000	168 → 170<br>169 → 171	0.68878<br>0.13124
430.85 nm	0.0000	168 → 170<br>169 → 171	-0.13031<br>0.68710
388.12 nm	0.0000	169 → 172	0.69372
380.12 nm	0.0619	165 → 170<br>167 → 170<br>169 → 173	-0.13704<br>0.62273<br>0.27563
368.62 nm	0.1320	165 → 170<br>167 →170<br>169 → 173	0.14693<br>-0.24118<br>0.63714

Cartesian coordinates of the optimized structure of 3aa

Lowest frequency = 14.100 cm^{-1}

Charge = 0, Multiplicity =1

S	1.595466	3.799286	-0.264109
O	-1.509543	2.519460	-0.303967
N	2.143458	-0.224691	-0.004681
C	3.225140	4.409948	-0.242932
H	3.384249	5.476480	-0.309511
C	4.172432	3.435077	-0.146023
H	5.234224	3.633025	-0.127265
C	3.603205	2.123922	-0.078608
C	2.203007	2.166307	-0.139236
C	1.446913	0.989559	-0.090818
C	0.082894	0.704268	-0.064679
C	-1.173794	1.362298	-0.141295
C	4.273850	0.857648	-0.006027
C	5.762797	0.826000	0.009264
C	6.460970	1.366778	1.094614
H	5.908661	1.791526	1.925285
C	7.853194	1.345122	1.123475
H	8.380247	1.758527	1.975921
C	8.565710	0.787346	0.063340

H	9.649271	0.767185	0.086076
C	7.877808	0.255615	-1.026056
H	8.424585	-0.178142	-1.855486
C	6.486208	0.276796	-1.054035
H	5.955524	-0.142610	-1.899590
C	3.527300	-0.299646	0.012007
C	4.179773	-1.635883	-0.003316
C	4.950044	-2.052685	1.082646
H	5.018674	-1.425243	1.962722
C	5.628950	-3.267479	1.035279
H	6.220816	-3.585624	1.885761
C	5.550353	-4.067629	-0.101906
H	6.082063	-5.011489	-0.140722
C	4.785378	-3.650812	-1.191472
H	4.720376	-4.269810	-2.078954
C	4.100890	-2.442377	-1.141580
H	3.503455	-2.120289	-1.986484
S	-1.595523	-3.799336	0.263953
O	1.509516	-2.519525	0.303539
N	-2.143468	0.224630	0.004302
C	-3.225214	-4.409962	0.243090
H	-3.384338	-5.476485	0.309772
C	-4.172500	-3.435075	0.146267

H	-5.234300	-3.633001	0.127703
C	-3.603255	-2.123938	0.078668
C	-2.203047	-2.166352	0.139058
C	-1.446933	-0.989625	0.090435
C	-0.082912	-0.704356	0.064089
C	1.173777	-1.362404	0.140555
C	-4.273885	-0.857653	0.006126
C	-5.762835	-0.825974	-0.008931
C	-6.461191	-1.366800	-1.094139
H	-5.909021	-1.791604	-1.924874
C	-7.853419	-1.345119	-1.122781
H	-8.380615	-1.758561	-1.975120
C	-8.565756	-0.787272	-0.062564
H	-9.649320	-0.767091	-0.085129
C	-7.877671	-0.255494	1.026694
H	-8.424308	0.178317	1.856188
C	-6.486067	-0.276700	1.054453
H	-5.955242	0.142741	1.899903
C	-3.527308	0.299620	-0.012133
C	-4.179726	1.635885	0.003162
C	-4.950217	2.052583	-1.082683
H	-5.019073	1.425030	-1.962663
C	-5.629056	3.267414	-1.035321

H	-6.221098	3.585476	-1.885711
C	-5.550164	4.067710	0.101741
H	-6.081821	5.011600	0.140553
C	-4.784959	3.651002	1.191187
H	-4.719726	4.270114	2.078572
C	-4.100542	2.442527	1.141300
H	-3.502929	2.120524	1.986110

References

[1] M. Grzybowski, E. Glodkowska-Mrowka, T. Stoklosa, D. T. Gryko, *Org. Lett.* **2012**, *14*, 2670–2673.

[2] a) R. Mei, S.-K. Zhang, L. Ackermann, *Synlett* **2017**, *28*, 1715–1718; b) M. J. Mio, L. C. Kopel, J. B. Braun, T. L. Gadzikwa, K. L. Hull, R. G. Brisbois, C. J. Markworth, P. A. Grieco, *Org. Lett.* **2002**, *4*, 3199–3202.

[3] M. J. Frisch, G. W. Trucks, H. B. Schlegel, G. E. Scuseria, M. A. Robb, J. R. Cheeseman, G. Scalmani, V. Barone, G. A. Petersson, H. Nakatsuji, X. Li, M. Caricato, A. V. Marenich, J. Bloino, B. G. Janesko, R. Gomperts, B. Mennucci, H. P. Hratchian, J. V. Ortiz, A. F. Izmaylov, J. L. Sonnenberg, Williams, F. Ding, F. Lipparini, F. Egidi, J. Goings, B. Peng, A. Petrone, T. Henderson, D. Ranasinghe, V. G. Zakrzewski, J. Gao, N. Rega, G. Zheng, W. Liang, M. Hada, M. Ehara, K. Toyota, R. Fukuda, J. Hasegawa, M. Ishida, T. Nakajima, Y. Honda, O. Kitao, H. Nakai, T. Vreven, K. Throssell, J. A. Montgomery Jr., J. E. Peralta, F. Ogliaro, M. J. Bearpark, J. J. Heyd, E. N. Brothers, K. N. Kudin, V. N. Staroverov, T. A. Keith, R. Kobayashi, J. Normand, K. Raghavachari, A. P. Rendell, J. C. Burant, S. S. Iyengar, J. Tomasi, M. Cossi, J. M. Millam, M. Klene, C. Adamo, R. Cammi, J. W. Ochterski, R. L. Martin, K. Morokuma, O. Farkas, J. B. Foresman, D. J. Fox, Rev. A.03 ed., Gaussian, Inc, Wallingford, CT, **2016**.

[4] a) A. D. Becke, *J. Chem. Phys.* **1993**, *98*, 5648–5652; b) A. D. Becke, *Phys. Rev. A* **1988**, *38*, 3098–3100; c) C. Lee, W. Yang, R. G. Parr, *Phys. Rev. B* **1988**, *37*, 785–789.

[5] S. Grimme, S. Ehrlich, L. Goerigk, *J. Comput. Chem.* **2011**, *32*, 1456–1465.

[6] a) G. W. Spitznagel, T. Clark, P. von Ragué Schleyer, W. J. Hehre, *J. Comput. Chem.* **1987**, *8*, 1109–1116; b) M. M. Francl, W. J. Pietro, W. J. Hehre, J. S. Binkley, M. S. Gordon, D. J. DeFrees, J. A. Pople, *J. Chem. Phys.* **1982**, *77*, 3654–3665; c) T. Clark, J. Chandrasekhar, G. W. Spitznagel, P. V. R. Schleyer, *J. Comput. Chem.* **1983**, *4*, 294–301; d) A. D. McLean, G. S. Chandler, *J. Chem. Phys.* **1980**, *72*, 5639–5648; e) R. Krishnan, J. S. Binkley, R. Seeger, J. A. Pople, *J. Chem. Phys.* **1980**, *72*, 650–654.

[7] A. V. Marenich, C. J. Cramer, D. G. Truhlar, *J. Phys. Chem. B* **2009**, *113*, 6378–6396.

[8] R. Dennington, T. A. Keith, J. M. Millam, 5 ed., Semichem Inc., Shawnee Mission, KS, **2009**.

NMR Spectra

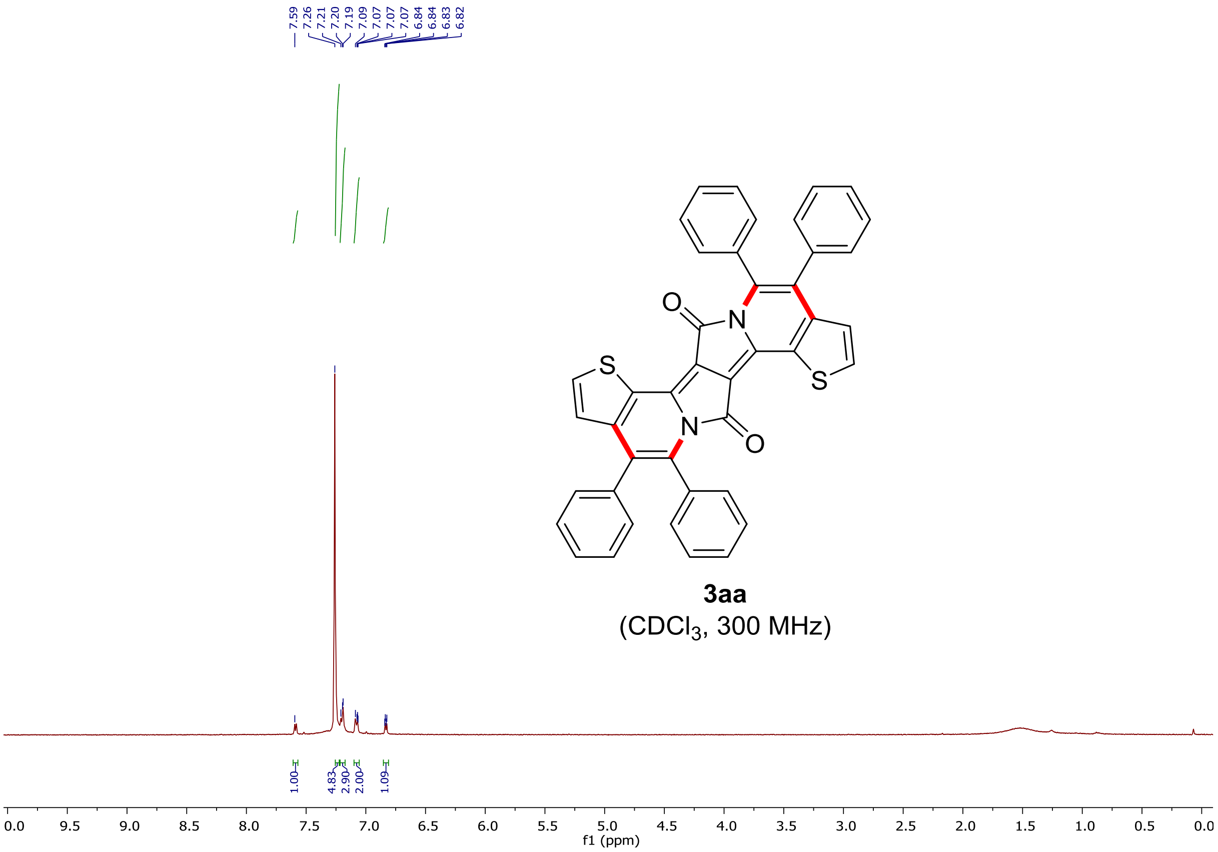

3aa
($CDCl_3$, 300 MHz)

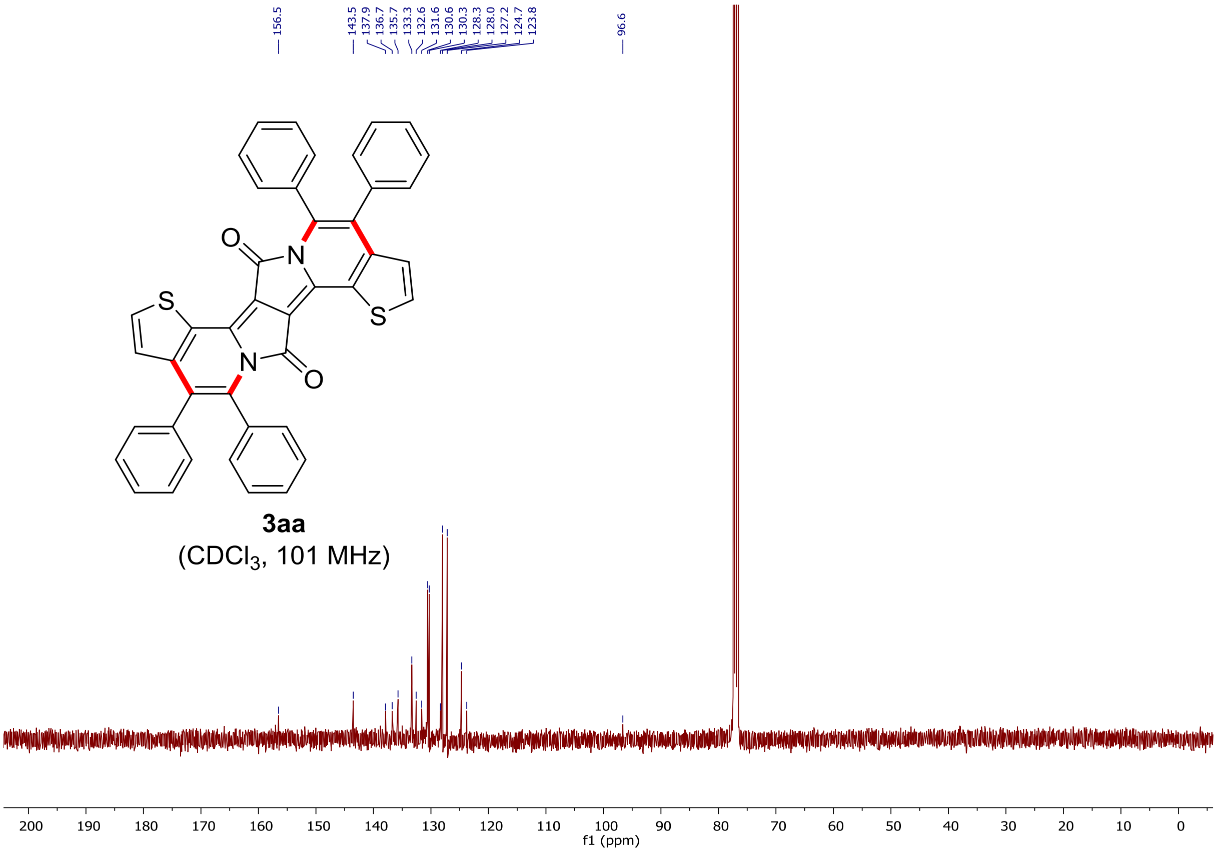

3aa
($CDCl_3$, 101 MHz)

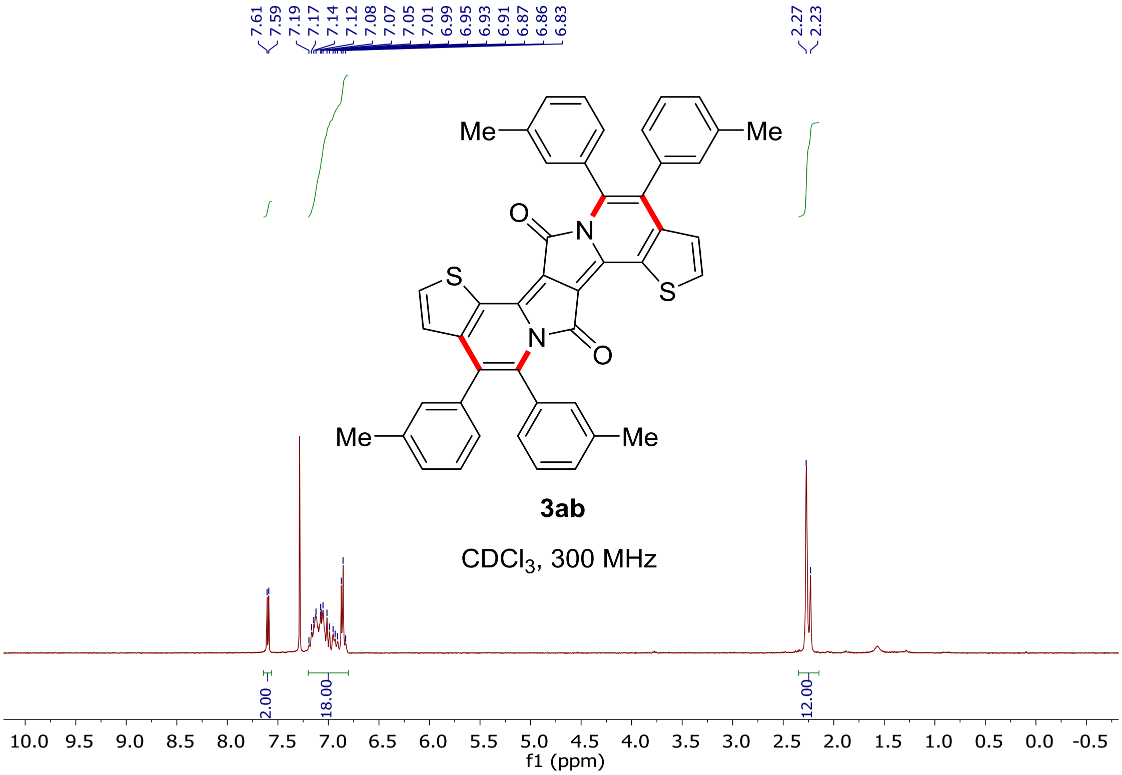
7.61
7.59
7.19
7.17
7.14
7.12
7.08
7.07
7.05
7.01
6.99
6.95
6.93
6.91
6.87
6.86
6.83
2.27
2.23
Me
Me
Me
Me
O
N
S
S
N
O
3ab
$CDCl_3$, 300 MHz
2.00
18.00
12.00
f1 (ppm)

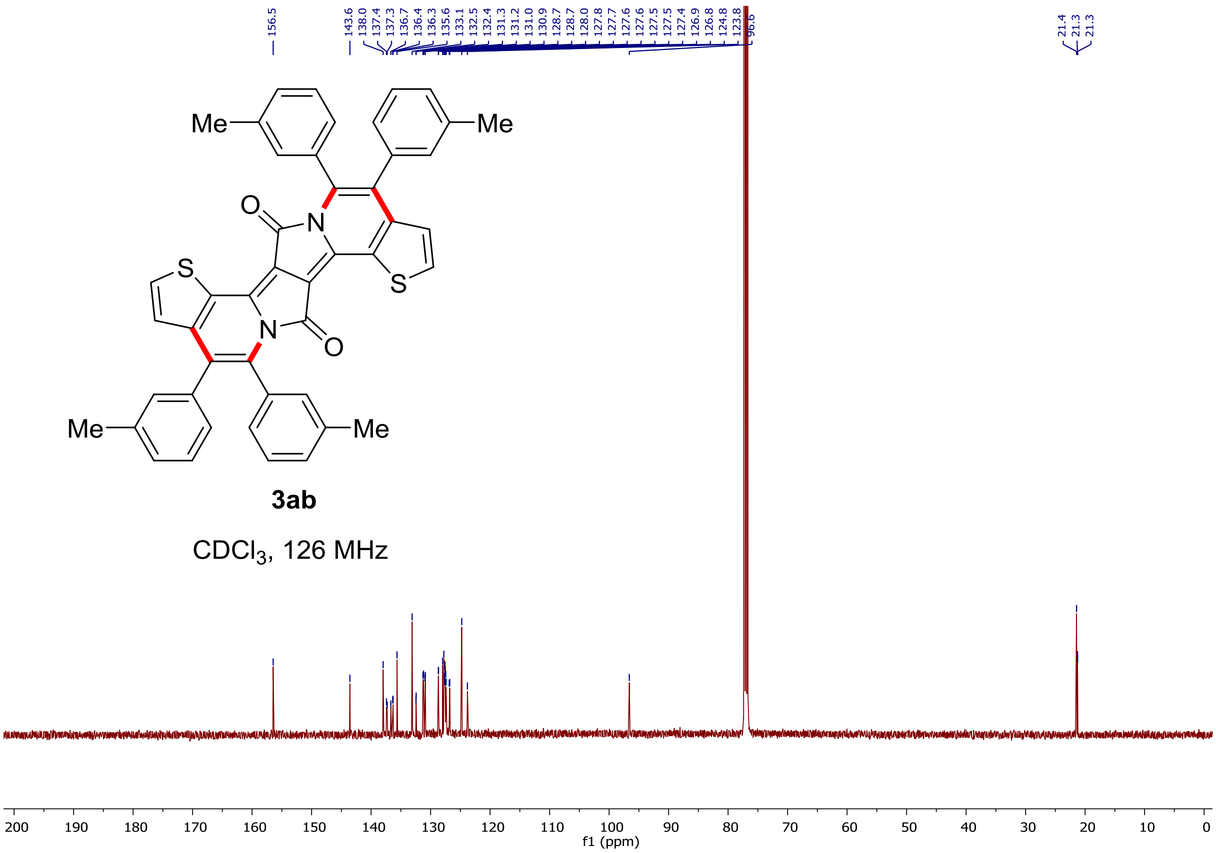
156.5
143.6
138.0
137.4
137.3
136.7
136.4
136.3
135.6
133.1
132.5
132.4
131.3
131.2
131.0
130.9
128.7
128.7
128.0
127.8
127.7
127.6
127.6
127.5
127.5
127.4
126.9
126.8
124.8
123.8
96.6
21.4
21.3
21.3
Me
Me
Me
Me
O
N
S
S
N
O
3ab
$CDCl_3$, 126 MHz
f1 (ppm)

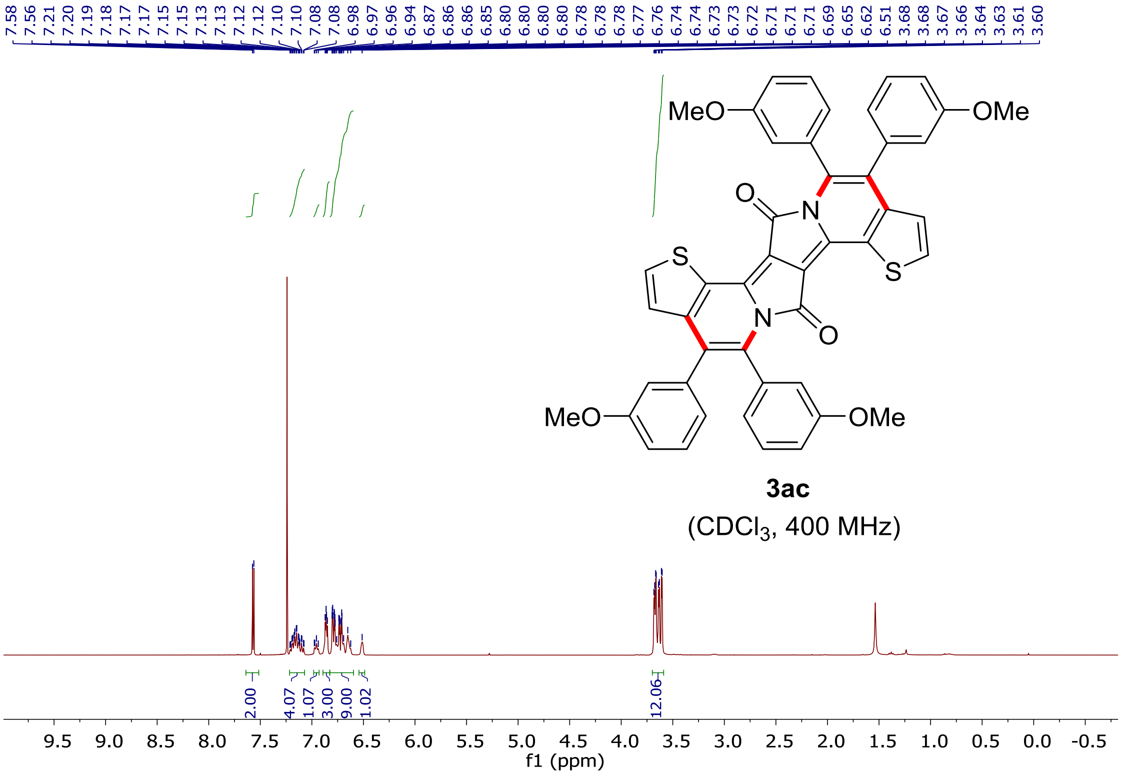

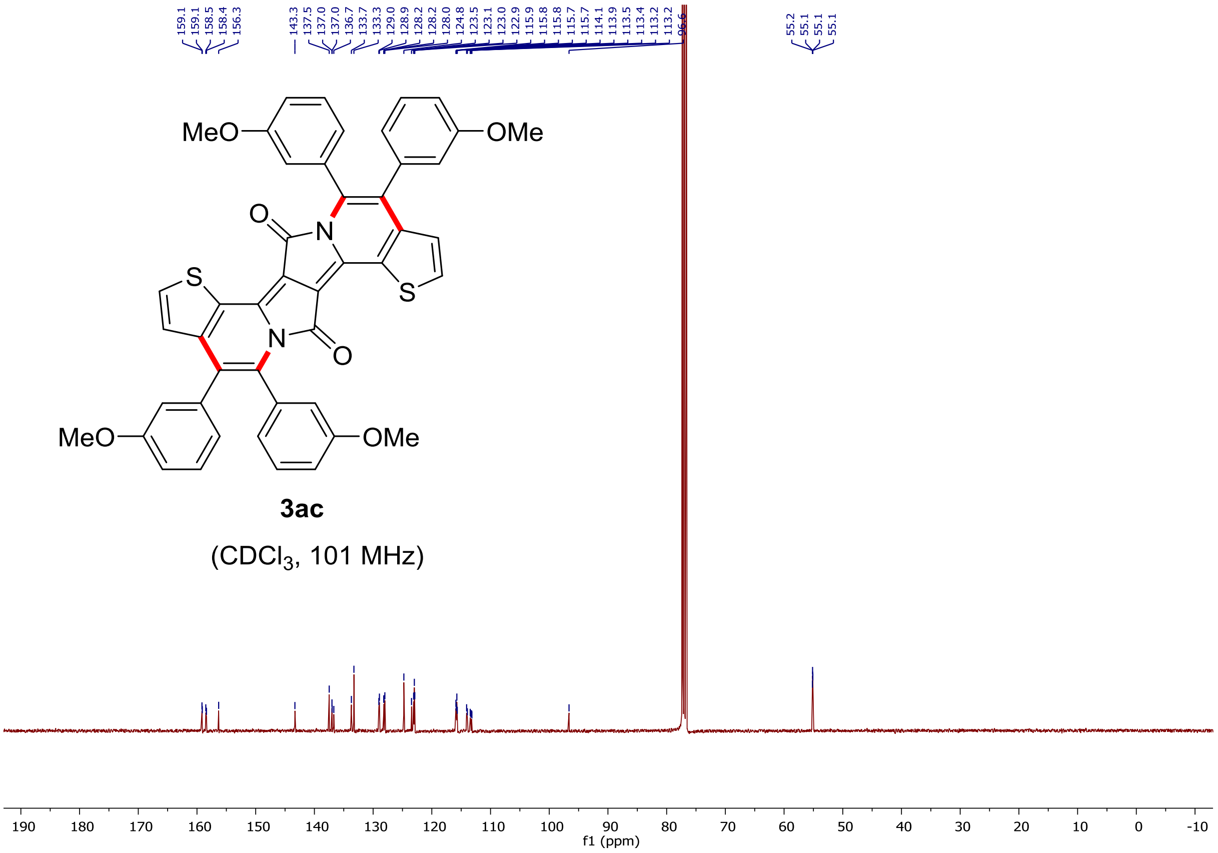

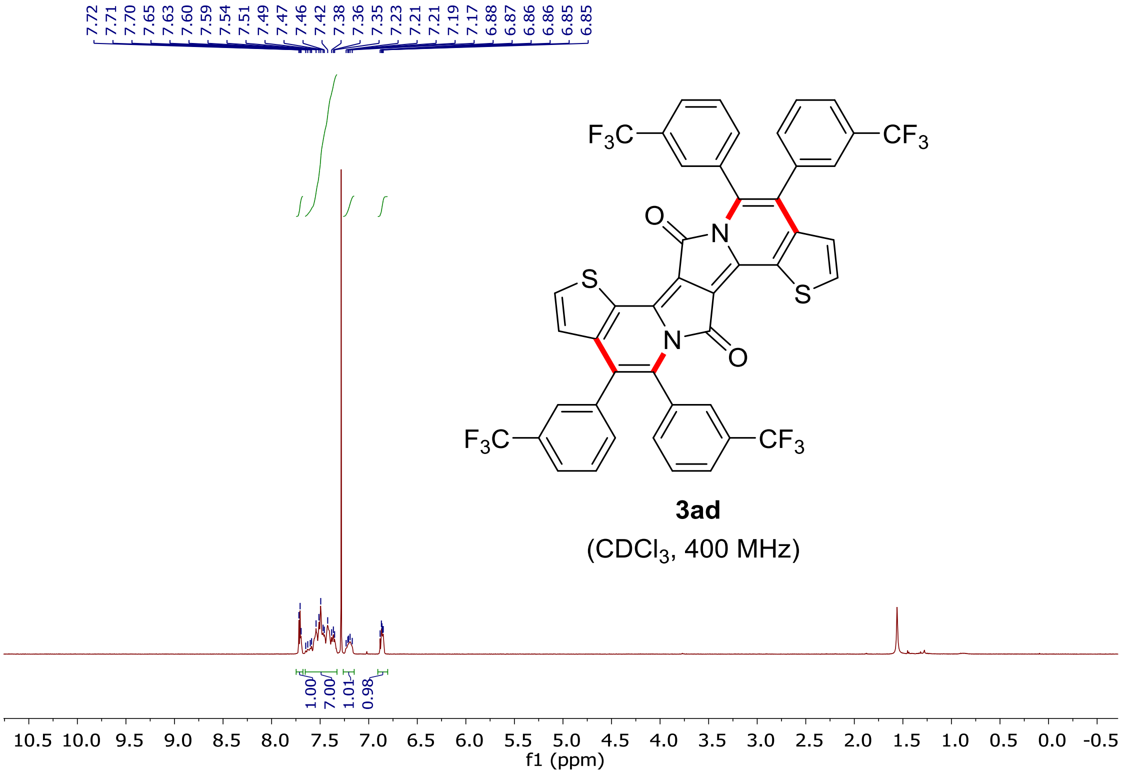

3ad

(CDCl$_3$, 400 MHz)

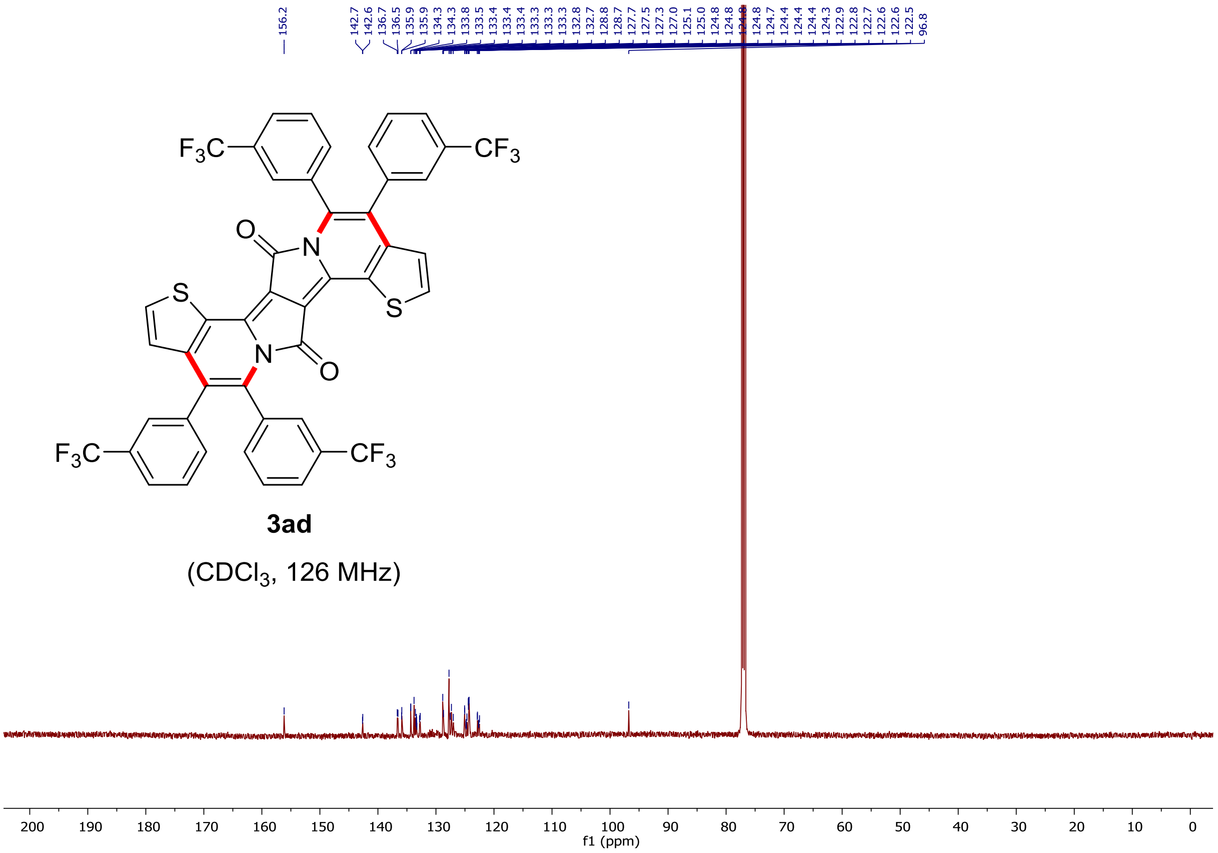

3ad

(CDCl$_3$, 126 MHz)

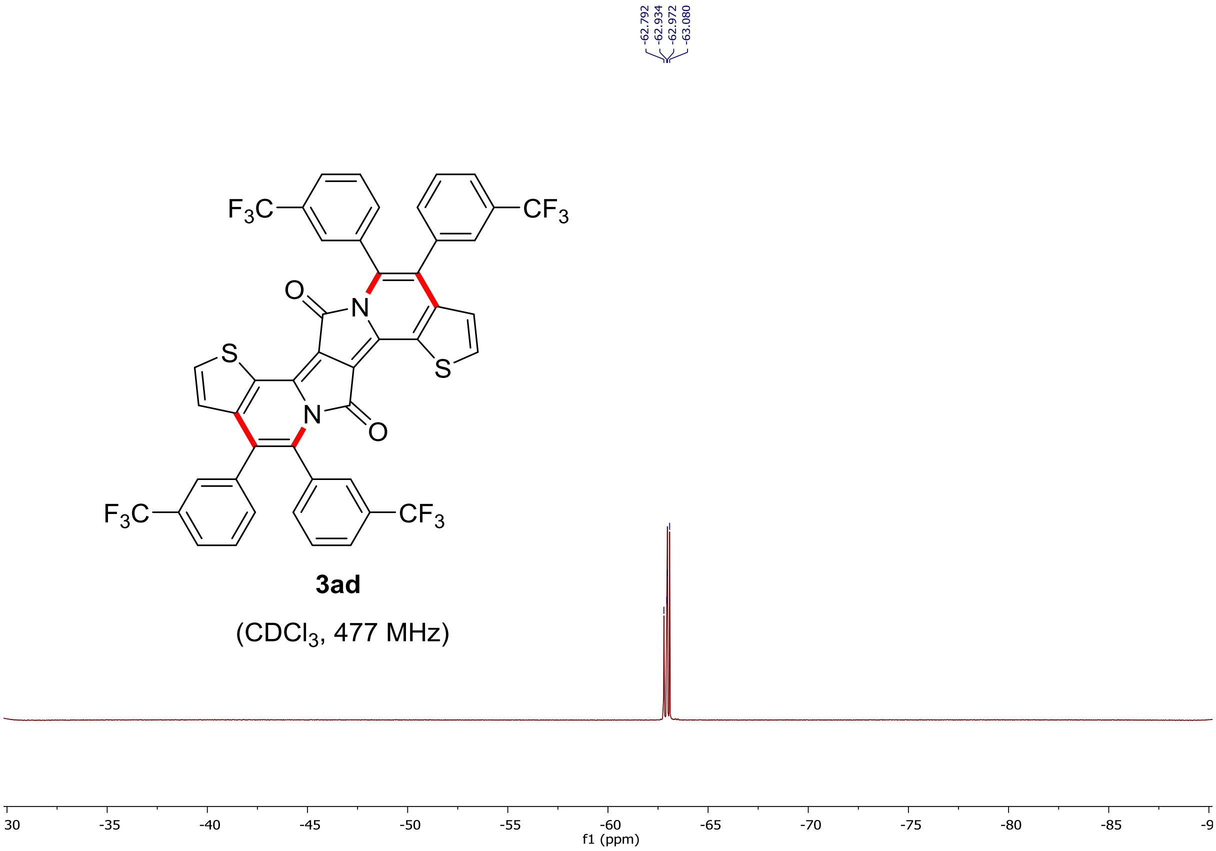
-62.792
-62.934
-62.972
-63.080
F3C
CF3
O
N
S
S
N
O
F3C
CF3
3ad
(CDCl3, 477 MHz)
30
-35
-40
-45
-50
-55
-60
-65
-70
-75
-80
-85
-9
f1 (ppm)

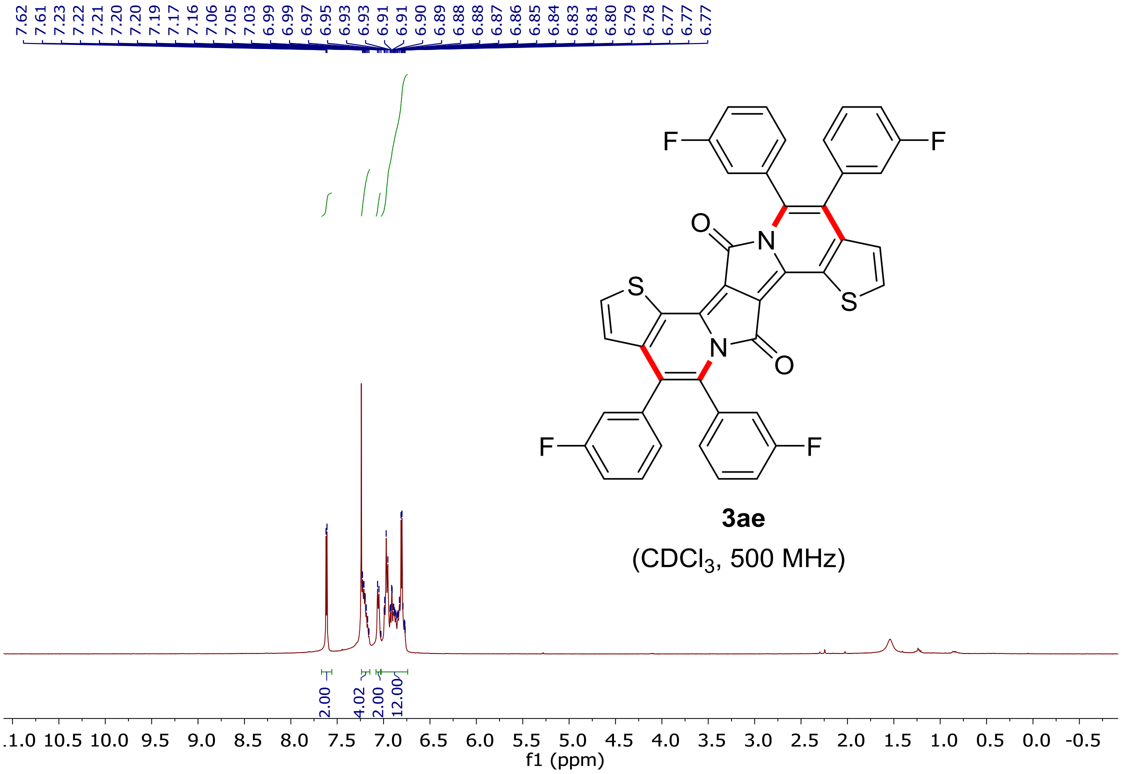
3ae
(CDCl3, 500 MHz)
f1 (ppm)

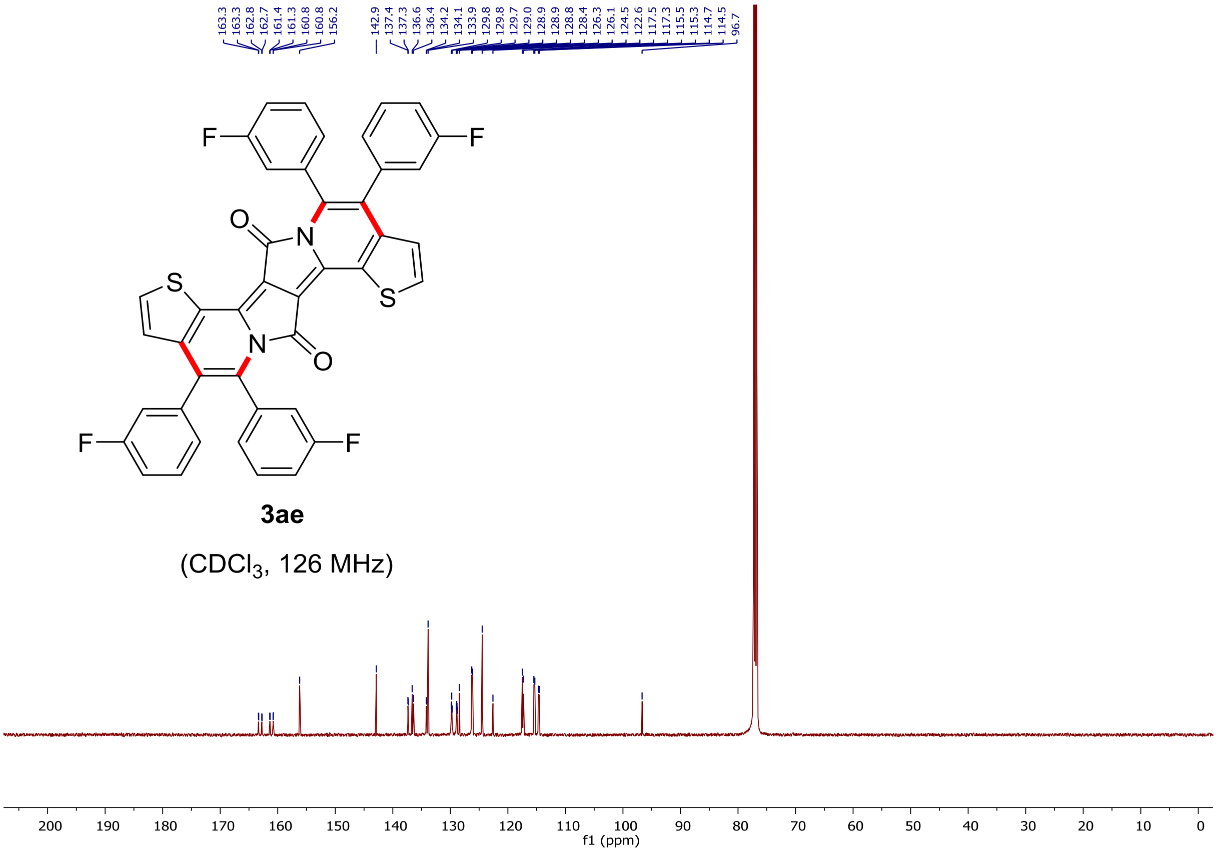
3ae
(CDCl3, 126 MHz)
f1 (ppm)

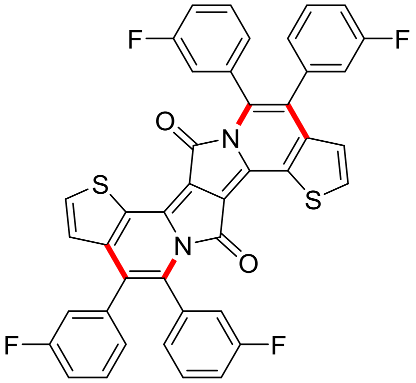

3ae

($CDCl_3$, 471 MHz)

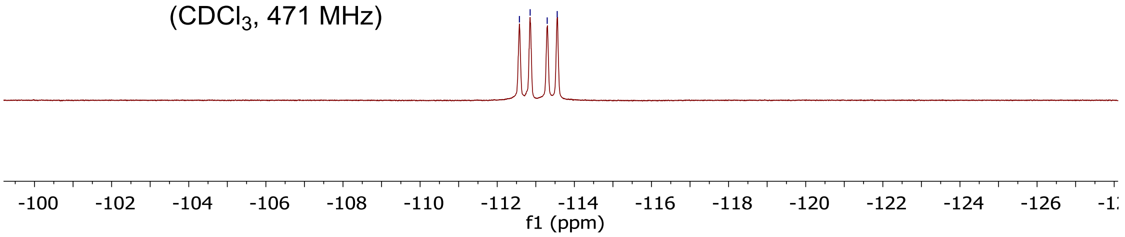

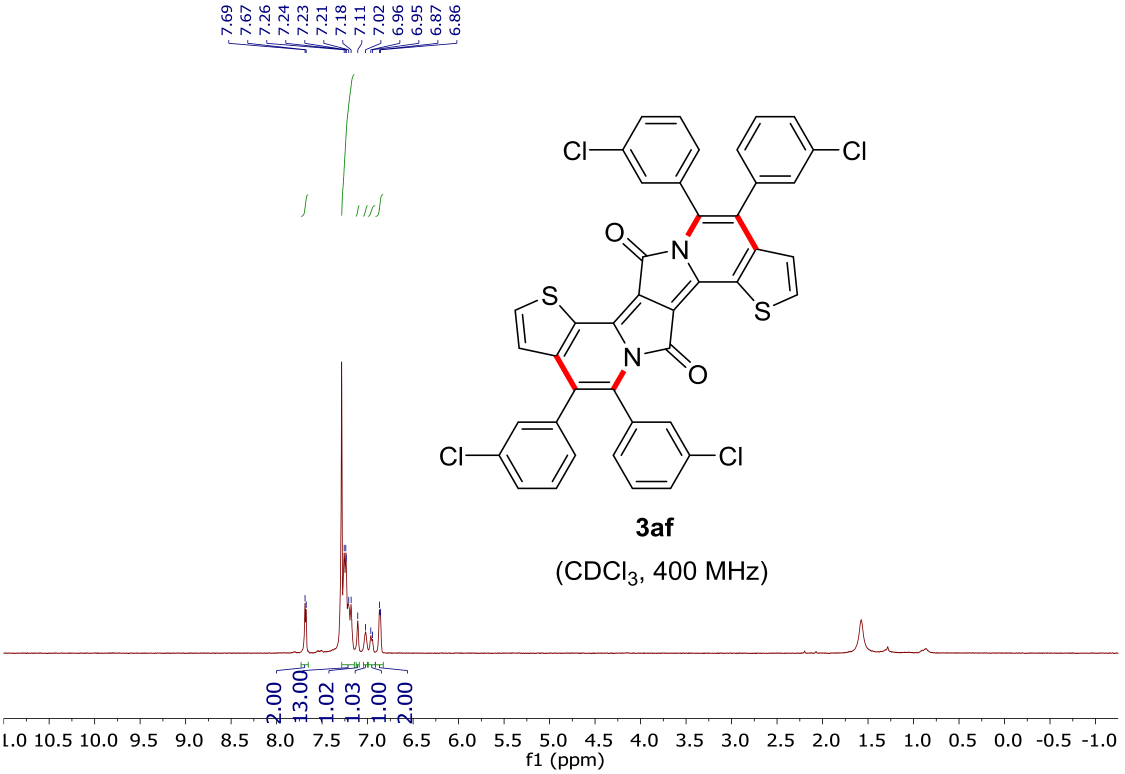
3af
(CDCl3, 400 MHz)
f1 (ppm)

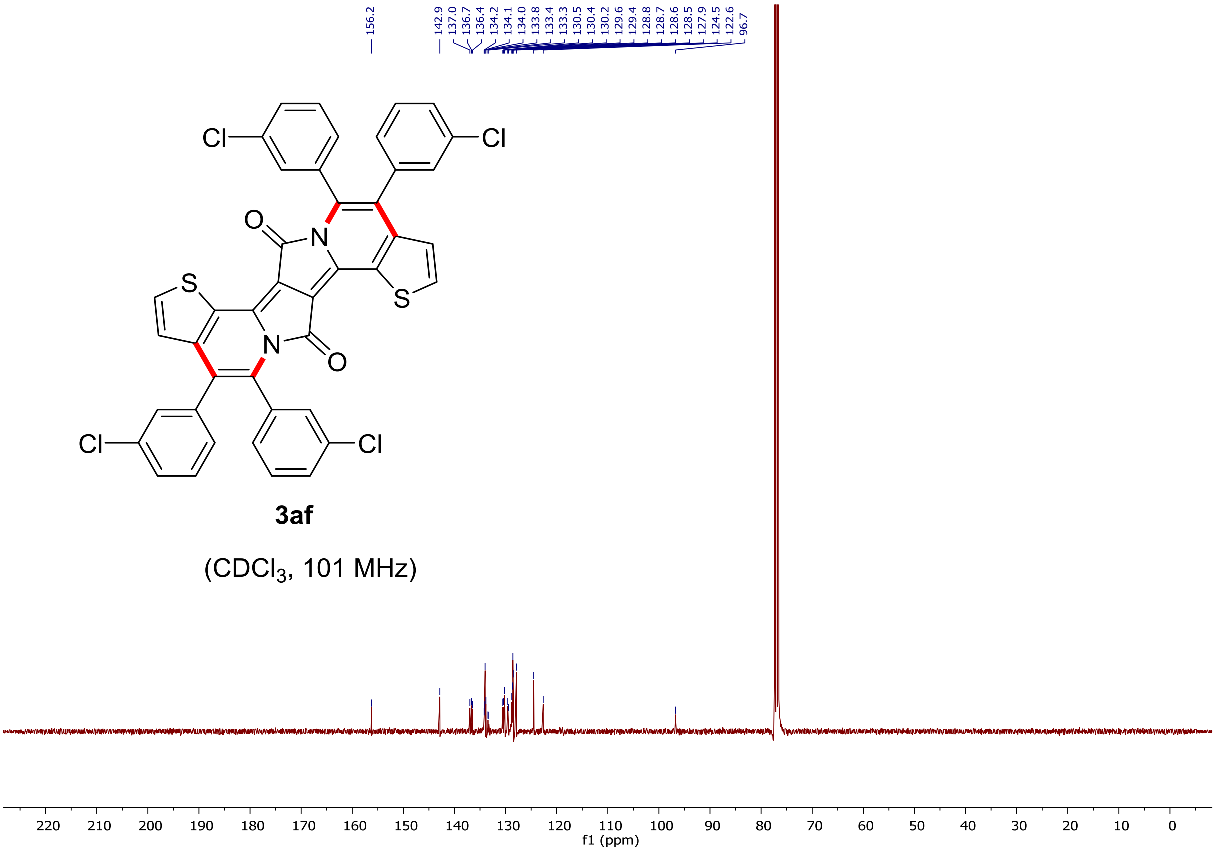
3af
(CDCl3, 101 MHz)
f1 (ppm)

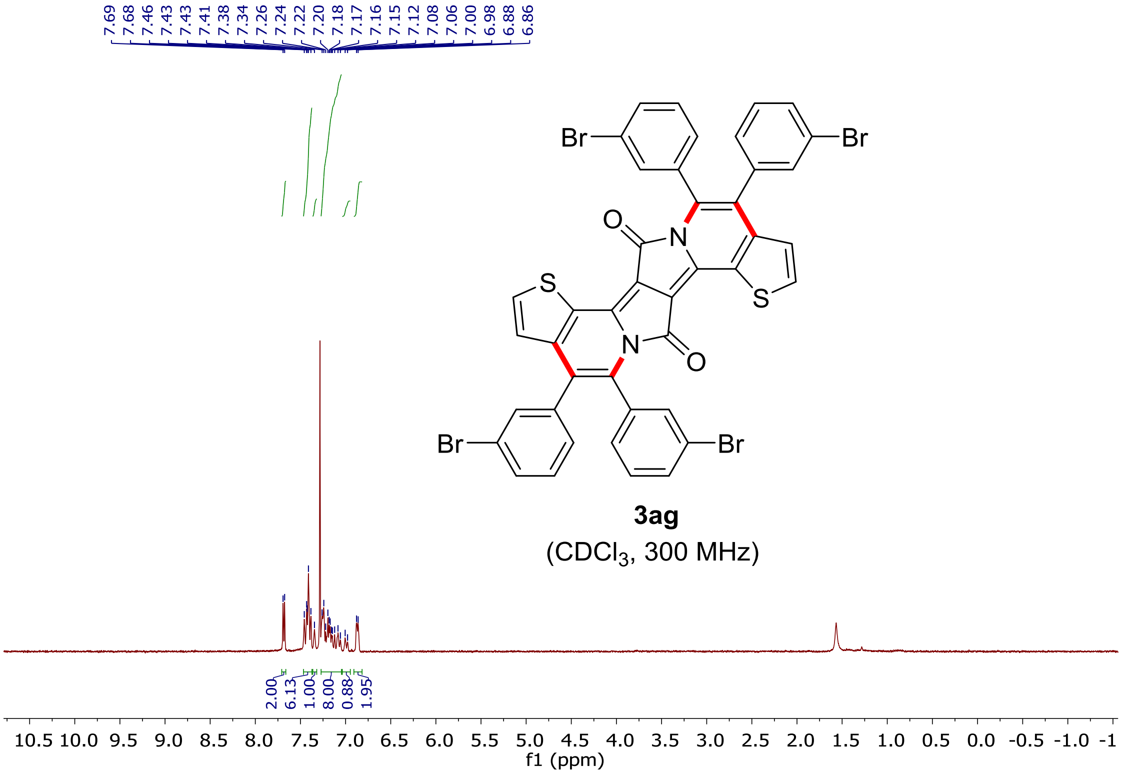
7.69
7.68
7.46
7.43
7.43
7.41
7.38
7.34
7.26
7.24
7.22
7.20
7.18
7.17
7.16
7.15
7.12
7.08
7.06
7.00
6.98
6.88
6.86
Br
Br
O
N
S
S
N
O
Br
Br
3ag
(CDCl3, 300 MHz)
2.00
6.13
1.00
8.00
0.88
1.95
f1 (ppm)

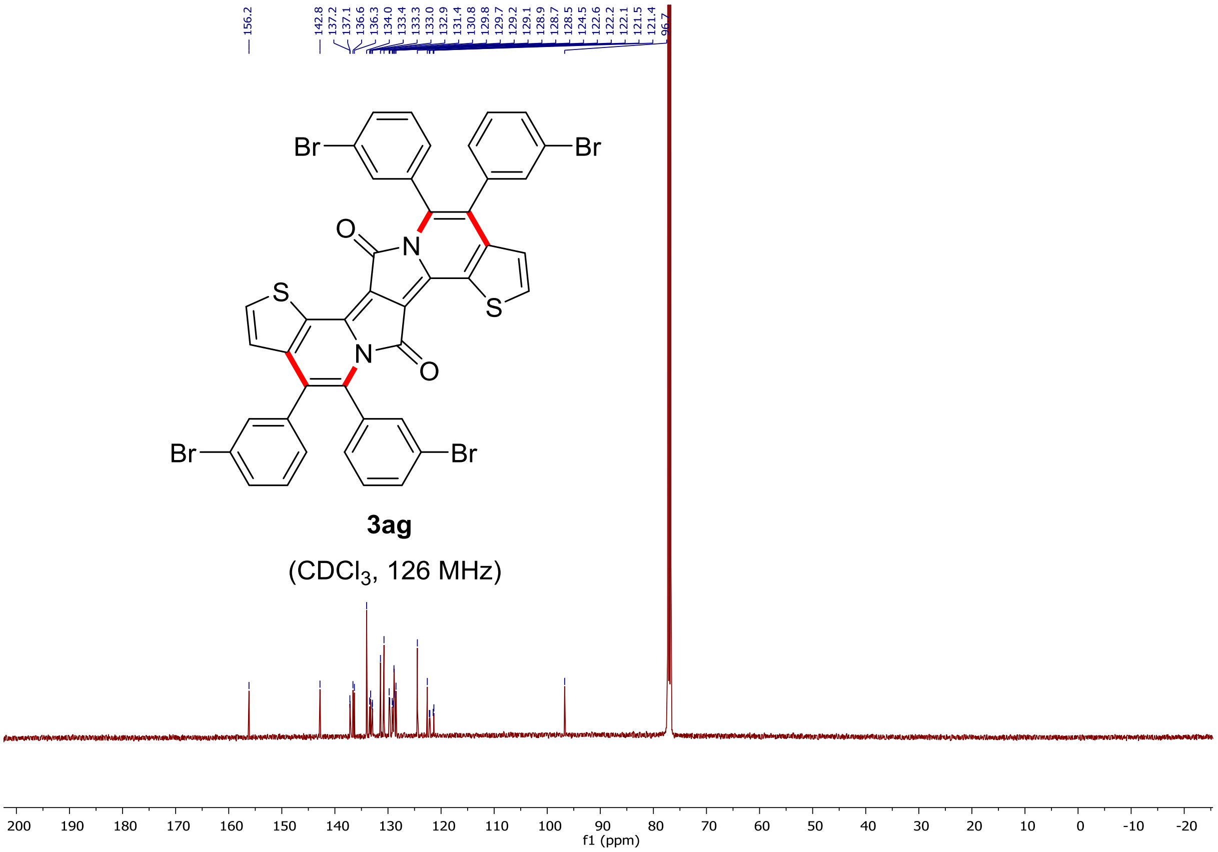
156.2
142.8
137.2
137.1
136.6
136.3
134.0
133.4
133.3
133.0
132.9
131.4
130.8
129.8
129.7
129.2
129.1
128.9
128.7
128.5
124.5
122.6
122.2
122.1
121.5
121.4
96.7
Br
Br
O
N
S
S
N
O
Br
Br
3ag
(CDCl3, 126 MHz)
f1 (ppm)

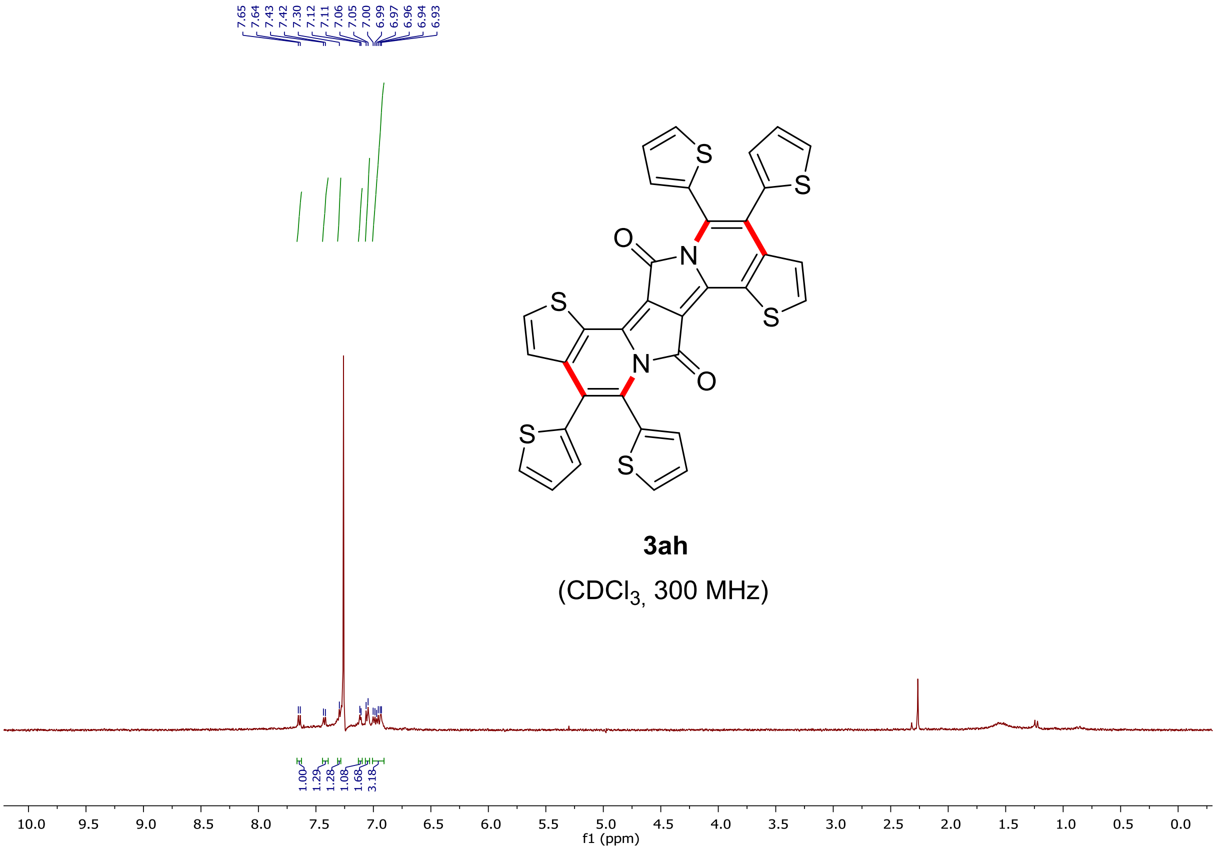
7.65
7.64
7.43
7.42
7.30
7.12
7.11
7.06
7.05
7.00
6.99
6.97
6.96
6.94
6.93
3ah
(CDCl3, 300 MHz)
1.00
1.29
1.28
1.08
1.68
3.18
10.0 9.5 9.0 8.5 8.0 7.5 7.0 6.5 6.0 5.5 5.0 4.5 4.0 3.5 3.0 2.5 2.0 1.5 1.0 0.5 0.0
f1 (ppm)

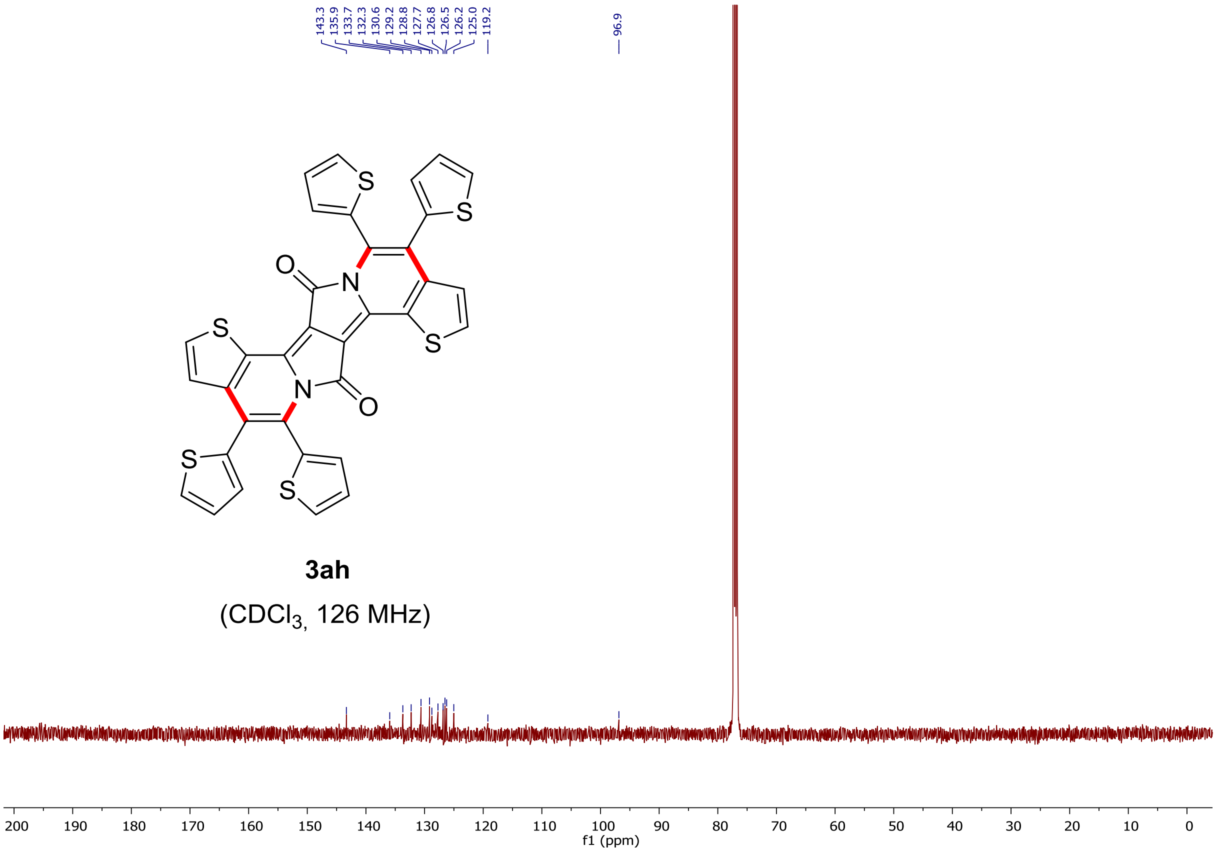
143.3
135.9
133.7
132.3
130.6
129.2
128.8
127.7
126.8
126.5
126.2
125.0
119.2
96.9
3ah
(CDCl3, 126 MHz)
200 190 180 170 160 150 140 130 120 110 100 90 80 70 60 50 40 30 20 10 0
f1 (ppm)

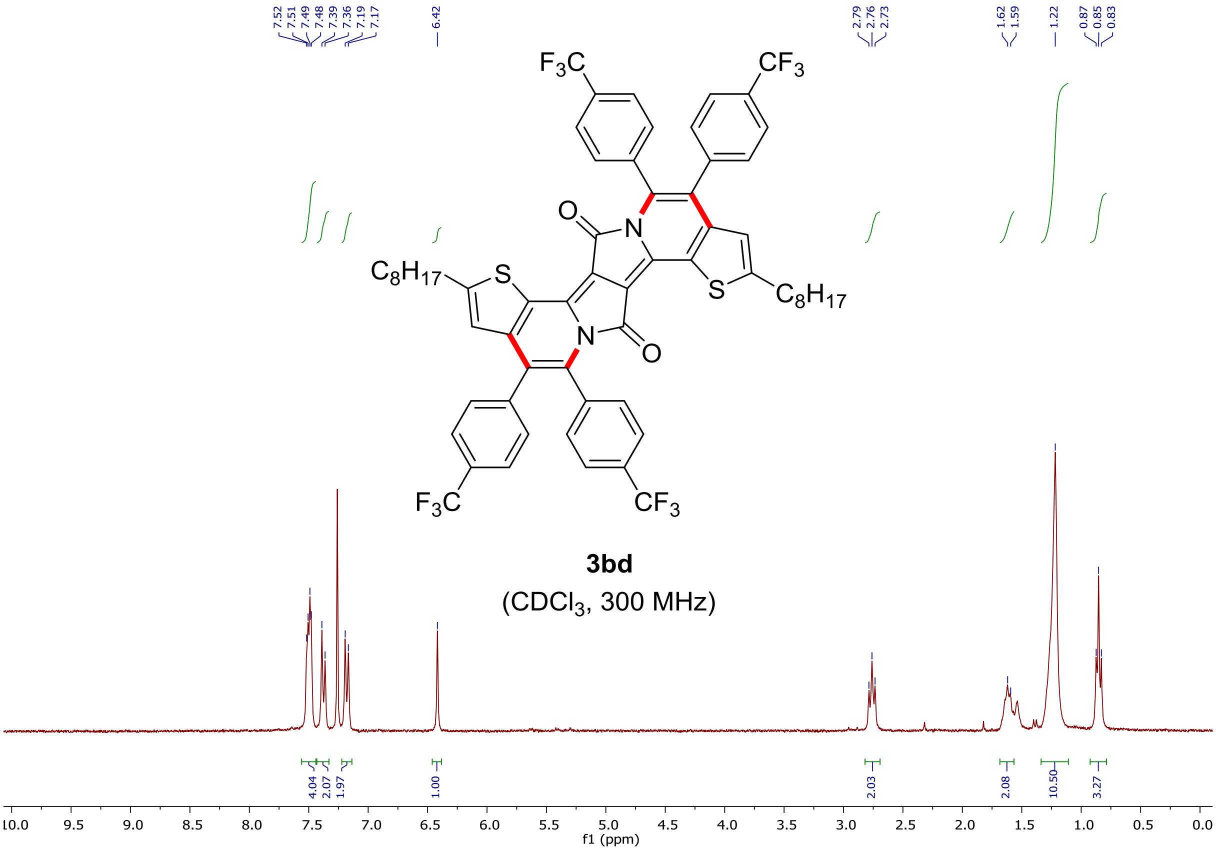
7.52 7.51 7.49 7.48 7.39 7.36 7.19 7.17
6.42
2.79 2.76 2.73
1.62 1.59
1.22
0.87 0.85 0.83
F3C
CF3
O
N
S
C8H17
3bd
(CDCl3, 300 MHz)
4.04 2.07 1.97
1.00
2.03
2.08
10.50
3.27
f1 (ppm)

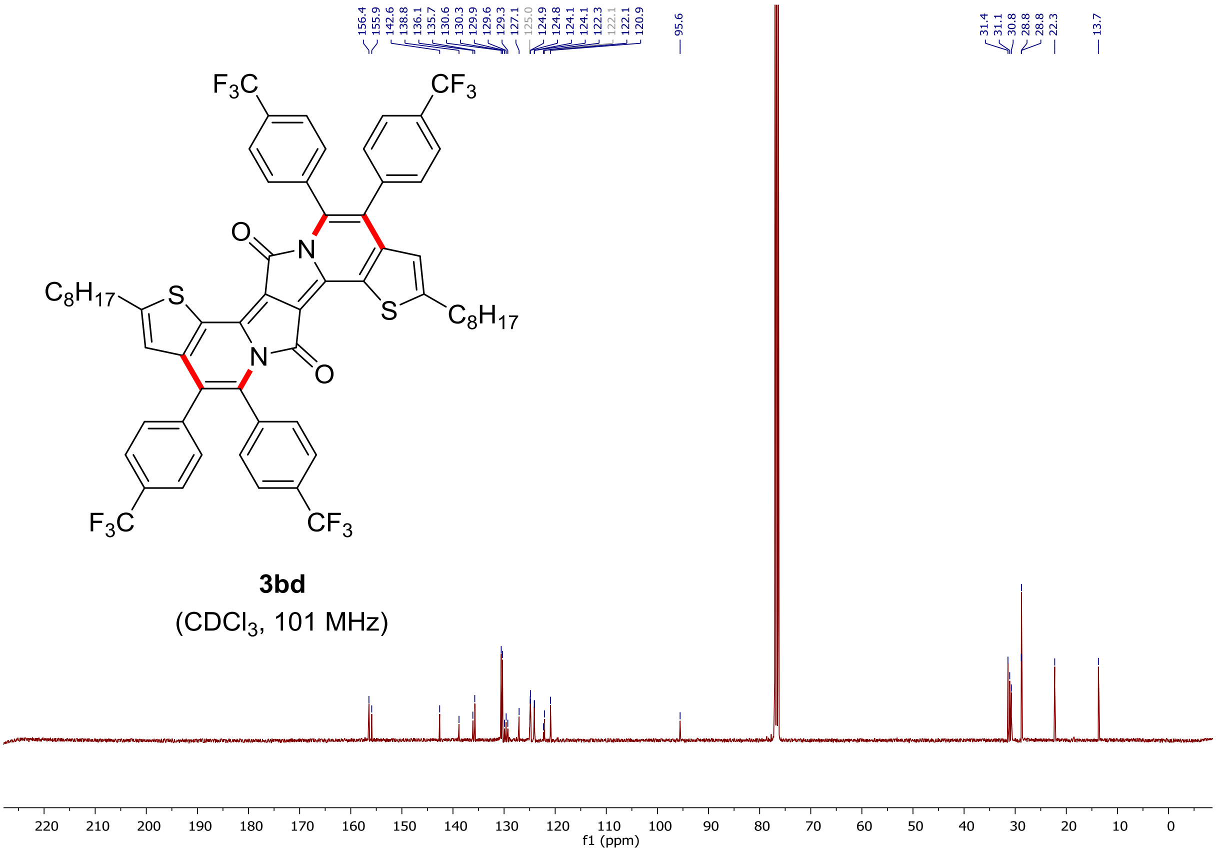
156.4 155.9 142.6 138.8 136.1 135.7 130.6 130.3 129.9 129.6 129.3 127.1 125.0 124.9 124.8 124.1 122.3 122.1 122.1 120.9
95.6
31.4 31.1 30.8 28.8 28.8 22.3
13.7
F3C
CF3
O
N
S
C8H17
3bd
(CDCl3, 101 MHz)
f1 (ppm)

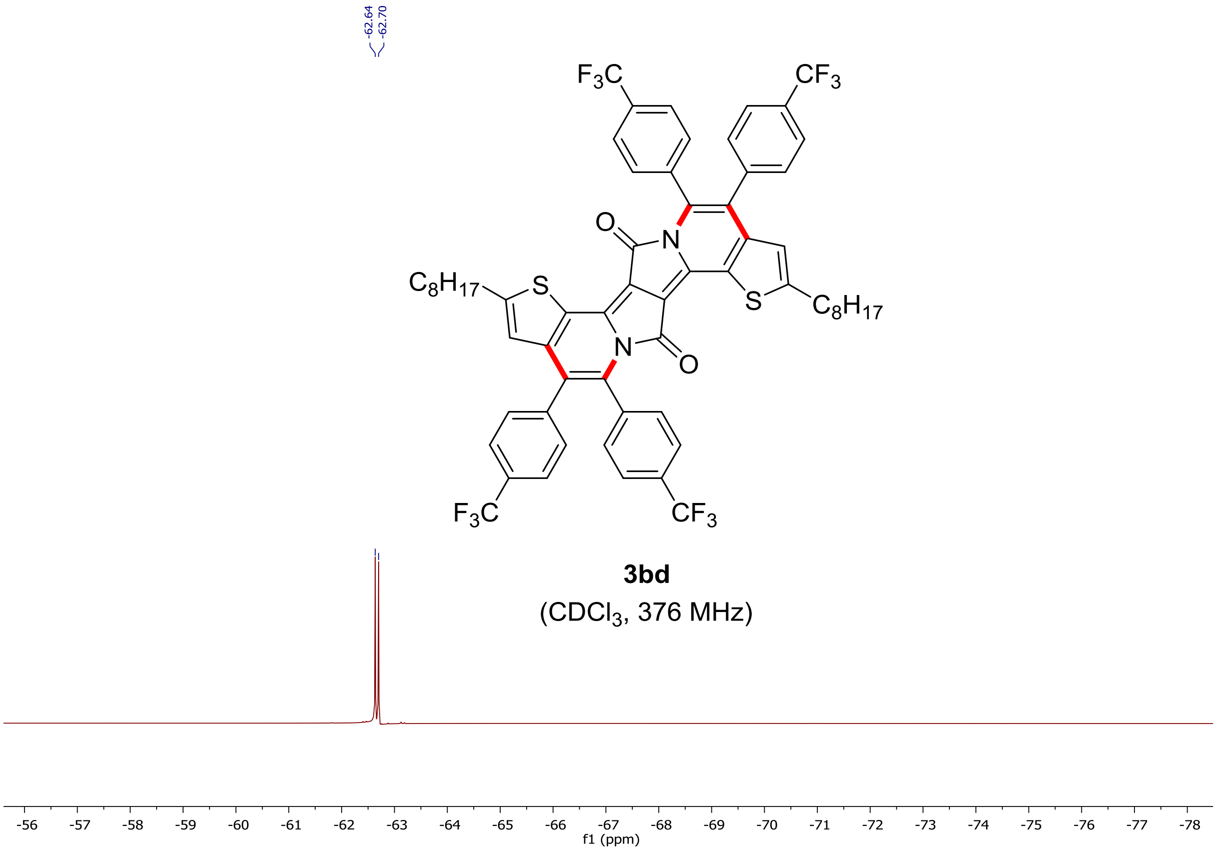

3bd

($CDCl_3$, 376 MHz)

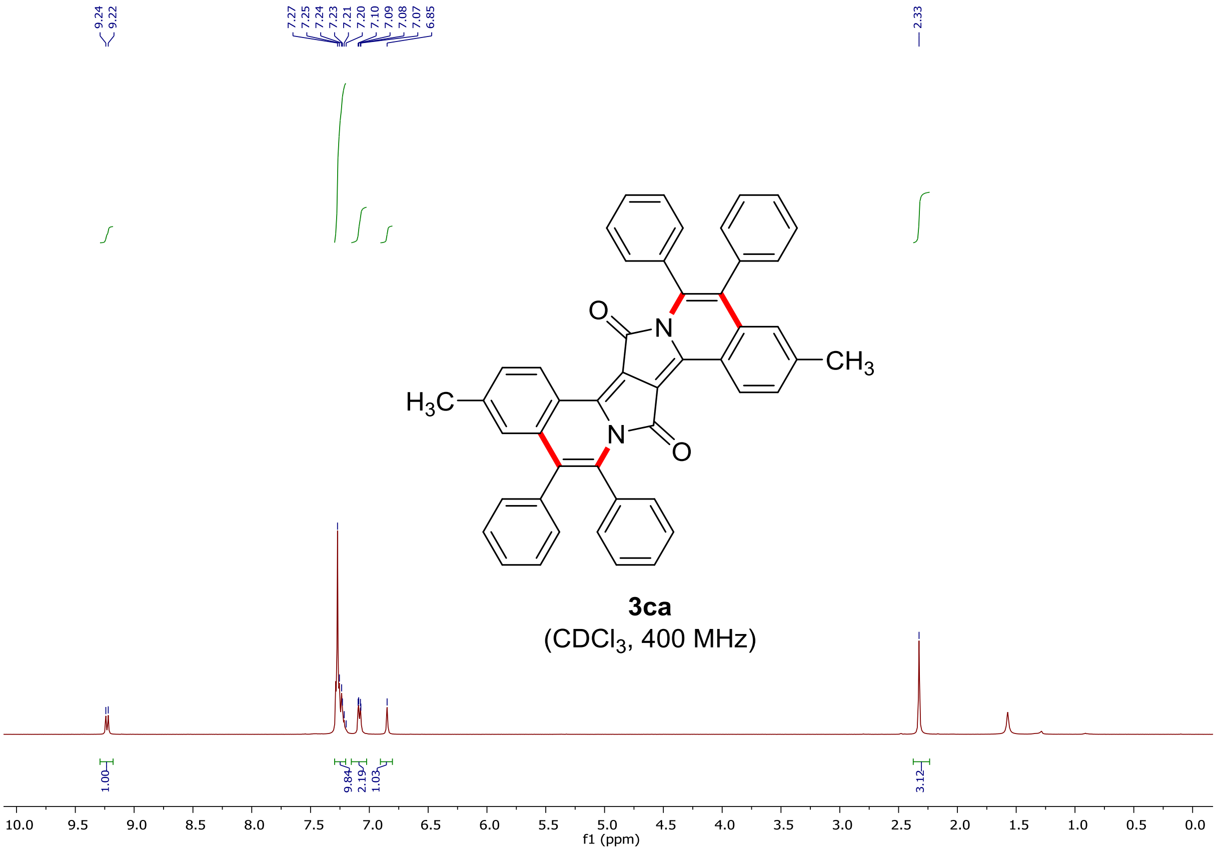
9.24
9.22
7.27
7.25
7.24
7.23
7.21
7.20
7.10
7.09
7.08
7.07
6.85
2.33
1.00
9.84
2.19
1.03
3.12
H3C
CH3
O
N
3ca
(CDCl3, 400 MHz)
f1 (ppm)

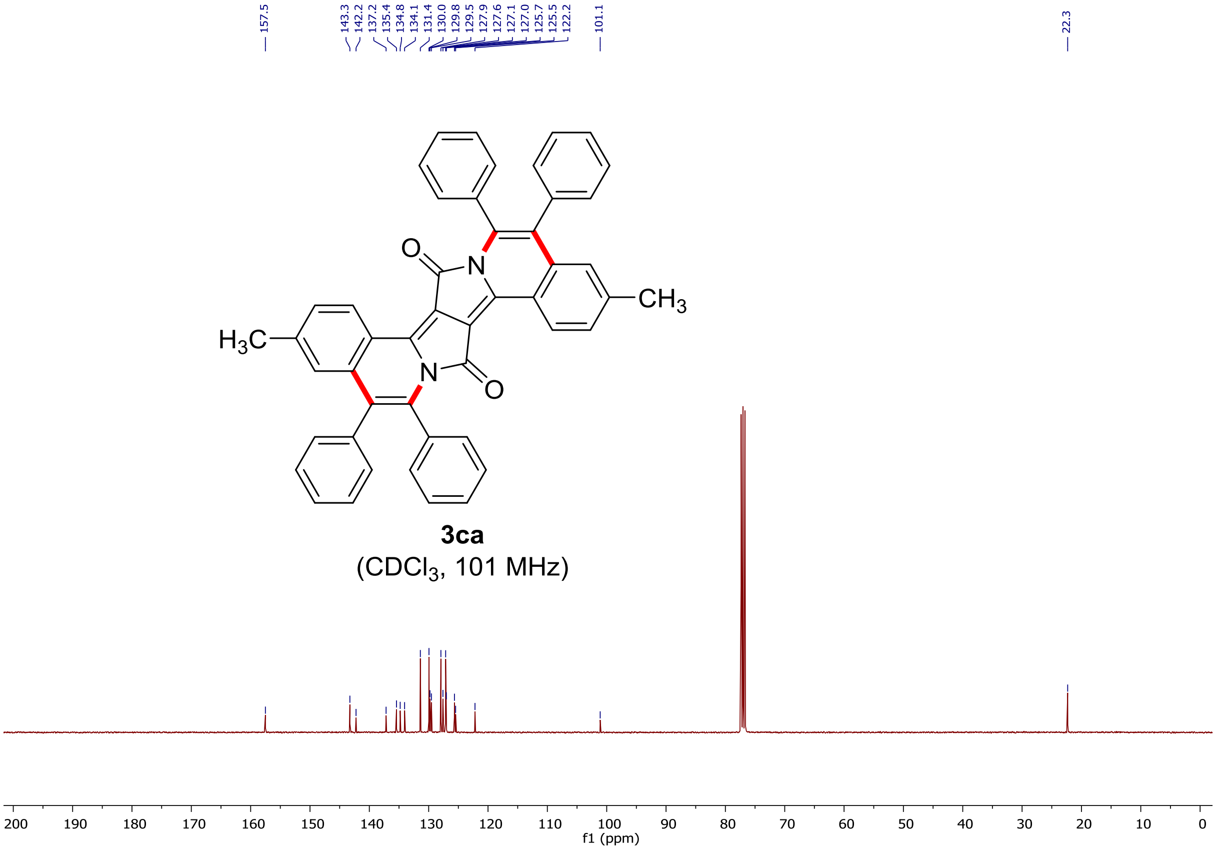
157.5
143.3
142.2
137.2
135.4
134.8
134.1
131.4
130.0
129.8
129.5
127.9
127.6
127.1
127.0
125.7
125.5
122.2
101.1
22.3
H3C
CH3
O
N
3ca
(CDCl3, 101 MHz)
f1 (ppm)

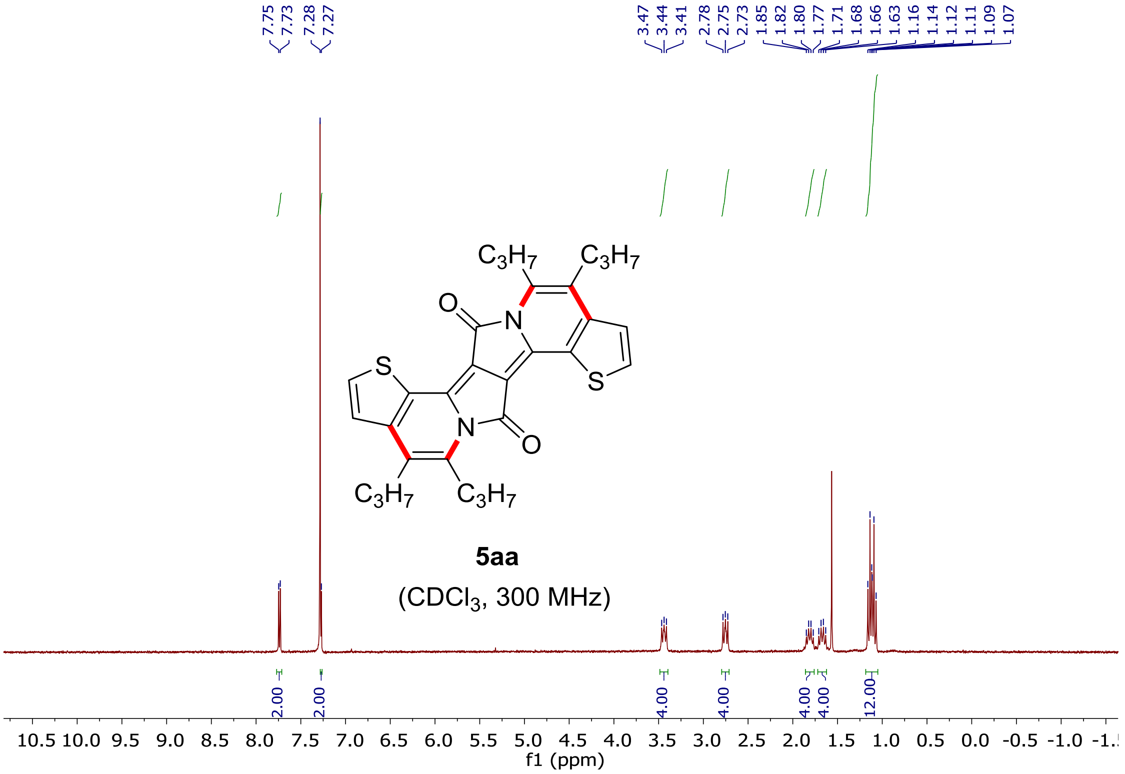
7.75
7.73
7.28
7.27
3.47
3.44
3.41
2.78
2.75
2.73
1.85
1.82
1.80
1.77
1.71
1.68
1.66
1.63
1.16
1.14
1.12
1.11
1.09
1.07
C_3H_7
C_3H_7
O
N
S
S
N
O
C_3H_7
C_3H_7
5aa
($CDCl_3$, 300 MHz)
2.00
2.00
4.00
4.00
4.00
4.00
12.00
10.5 10.0 9.5 9.0 8.5 8.0 7.5 7.0 6.5 6.0 5.5 5.0 4.5 4.0 3.5 3.0 2.5 2.0 1.5 1.0 0.5 0.0 -0.5 -1.0
f1 (ppm)

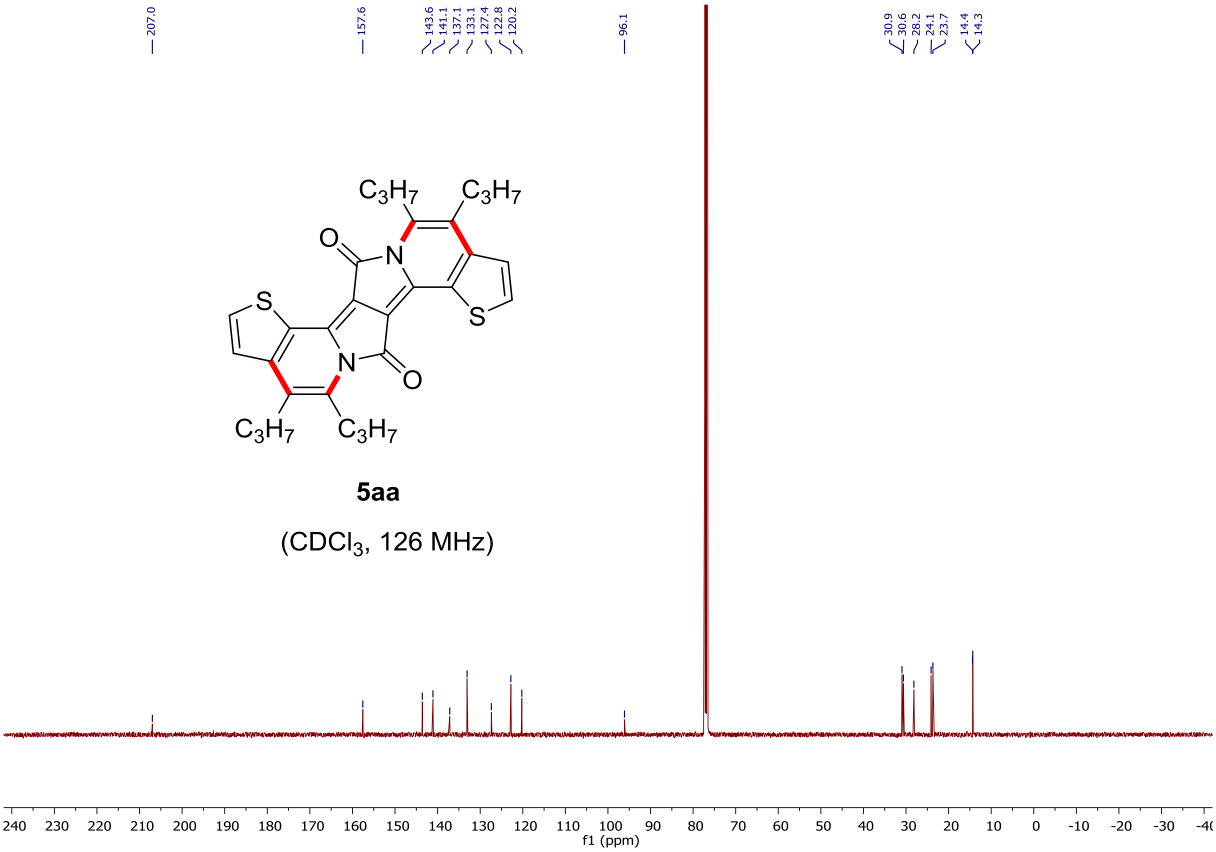
207.0
157.6
143.6
141.1
137.1
133.1
127.4
122.8
120.2
96.1
30.9
30.6
28.2
24.1
23.7
14.4
14.3
C_3H_7
C_3H_7
O
N
S
S
N
O
C_3H_7
C_3H_7
5aa
($CDCl_3$, 126 MHz)
240 230 220 210 200 190 180 170 160 150 140 130 120 110 100 90 80 70 60 50 40 30 20 10 0 -10 -20 -30
f1 (ppm)

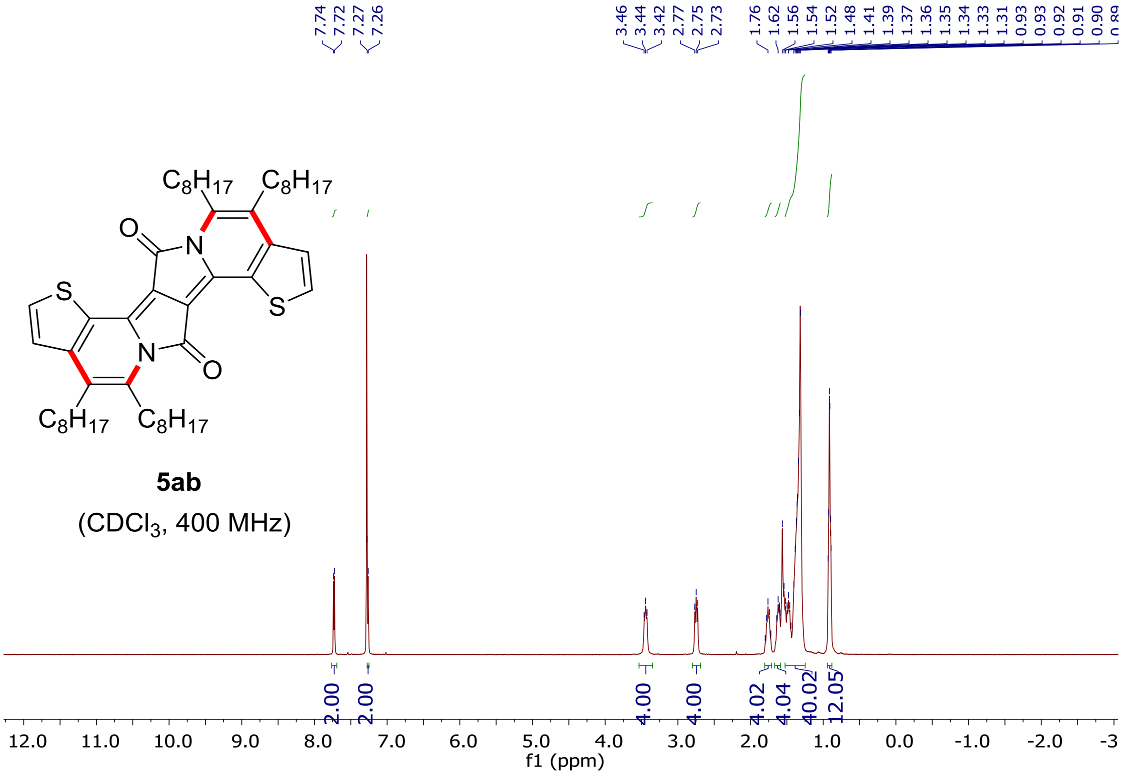
7.74
7.72
7.27
7.26
3.46
3.44
3.42
2.77
2.75
2.73
1.76
1.62
1.56
1.54
1.52
1.48
1.41
1.39
1.37
1.36
1.35
1.34
1.33
1.31
0.93
0.93
0.92
0.91
0.90
5ab
(CDCl3, 400 MHz)
2.00
2.00
4.00
4.00
4.02
4.04
40.02
12.05
f1 (ppm)

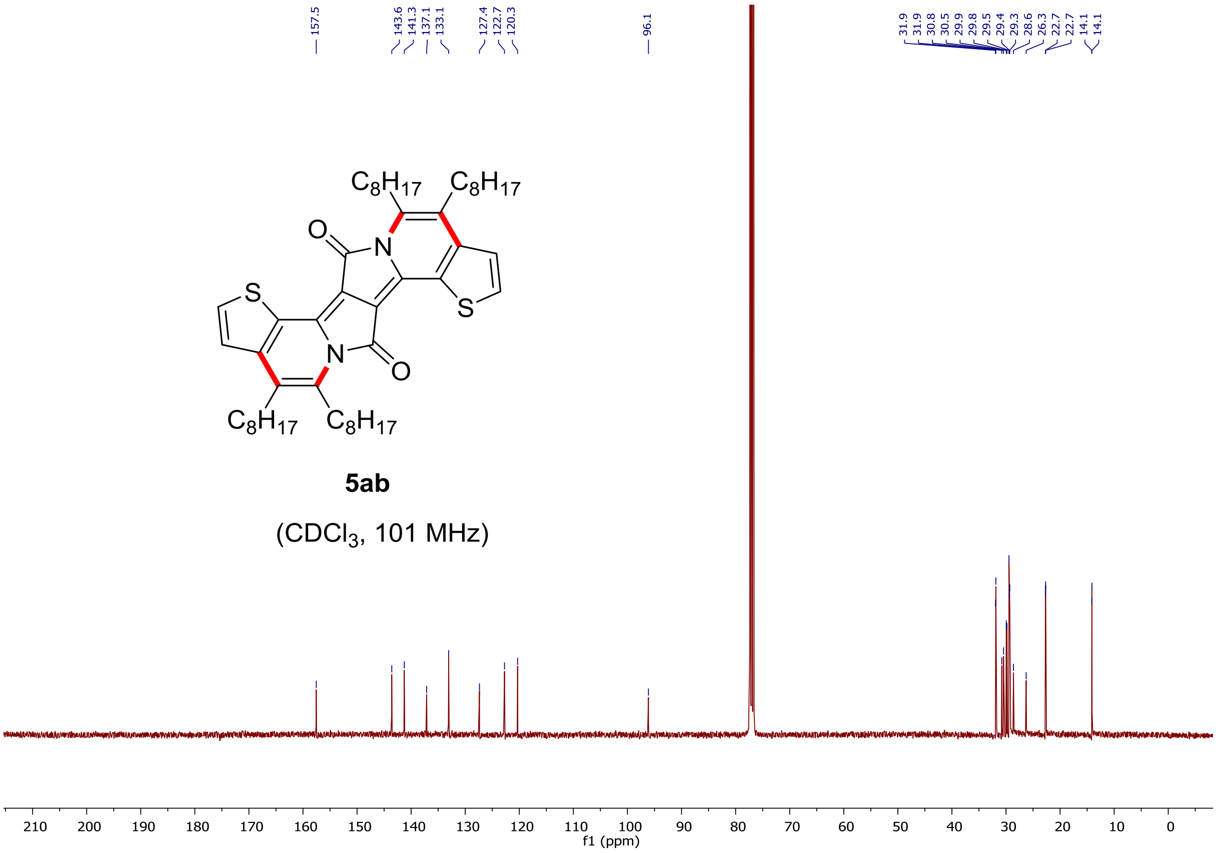
157.5
143.6
141.3
137.1
133.1
127.4
122.7
120.3
96.1
31.9
31.9
30.8
30.5
29.9
29.8
29.5
29.4
29.3
28.6
26.3
22.7
22.7
14.1
14.1
5ab
(CDCl3, 101 MHz)
f1 (ppm)

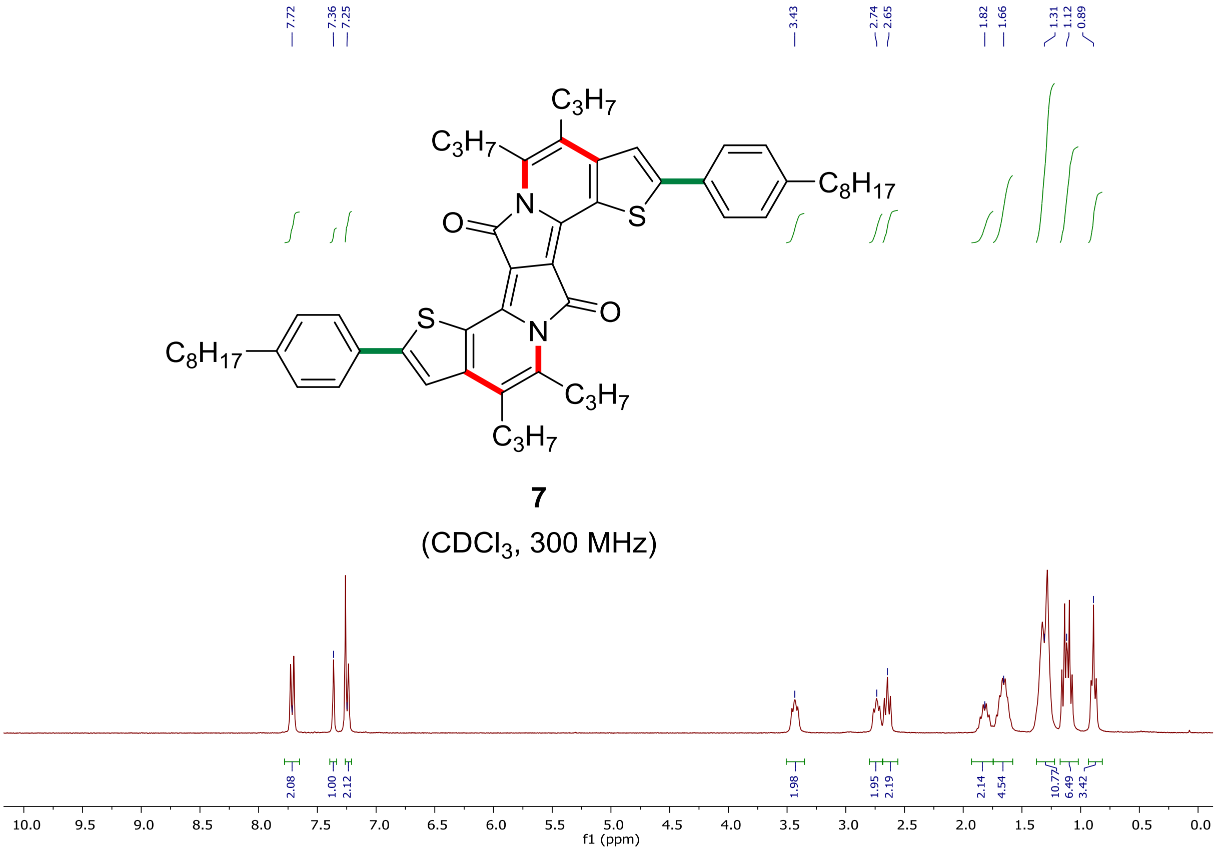

7

($CDCl_3$, 300 MHz)

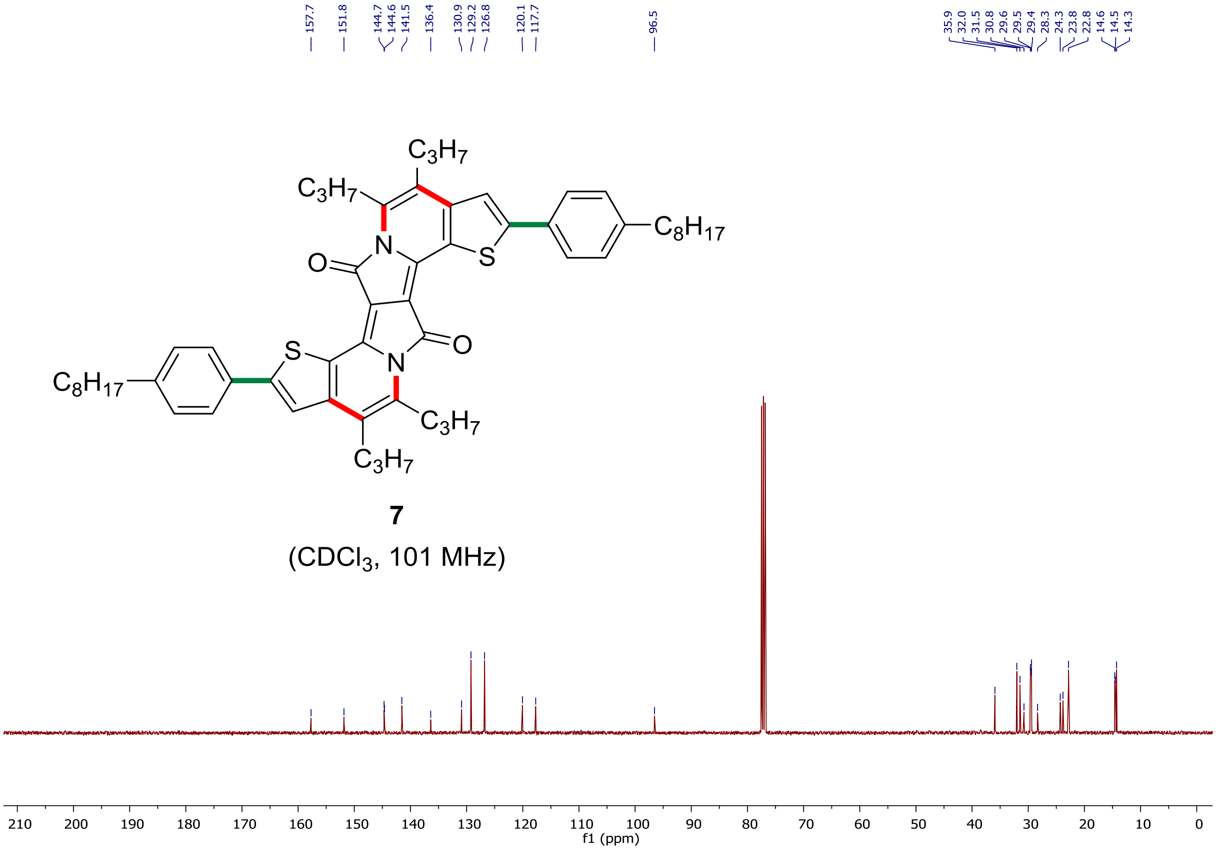

7

($CDCl_3$, 101 MHz)
